# Determination of the absolute configurations of the stereogenic centers of ustilaginoidins by studying the biosynthetic monomers from a gene knockout mutant of *Villosiclava virens*

**DOI:** 10.1038/s41598-018-37941-5

**Published:** 2019-02-12

**Authors:** Daowan Lai, Jiajia Meng, Dan Xu, Xuping Zhang, Yafeng Liang, Yu Han, Cong Jiang, Huiquan Liu, Chenfang Wang, Ligang Zhou, Jin-Rong Xu

**Affiliations:** 1 Department of Plant Pathology, College of Plant Protection, China Agricultural University, Beijing, 100193 China; 2 Department of Plant Pathology, College of Plant Protection, Northwest A&F University, Yangling, 712100 China; 3 Department of Botany and Plant Pathology, Purdue University, West Lafayette, IN 47907 United States

## Abstract

Ustilaginoidins are a kind of mycotoxins with 9,9′-linked bis-naphtho-γ-pyrones structures produced by the rice false smut pathogen *Villosiclava virens*. These metabolites displayed a wide range of bioactivities, such as teratogenic, cytotoxic, phytotoxic, and antibacterial activities. So far 26 ustilaginoidins have been isolated from *V*. *virens*, among which 18 compounds contained stereogenic center(s), however, most of them were unknown for the absolute configurations, except that of ustilaginoidin D. In this study, the absolute structures of these ustilaginoidins were constructed for the first time by analysis of the biosynthetic monomers obtained from a gene knockout mutant (Δ*UV_2091*) of *V*. *virens*. The gene *UV_2091* was predicted to encode an enzyme that dimerized the monomeric naphtho-γ-pyrones in *V*. *virens*. Knockout of this gene led to the accumulation of three monomers, namely hemiustilaginoidin F (**1**), epihemiustilaginoidin D (**2**), and hemiustilaginoidin D (**3**), but the production of ustilaginoidins was completely blocked. The structures of the monomers were deduced by spectroscopic analysis, in combination with TDDFT ECD calculations for determining the absolute configurations. These compounds were tested for their phytotoxic, cytotoxic, antibacterial, and antifungal activities. Compounds **1** and **3** showed inhibition against the radicle and plumule elongation of rice and lettuce seeds at the tested concentrations. Compound **1** was active against the tested five human cancer cells, with half maximal inhibitory concentrations (IC_50_s) of 13.2~37.3 μM. Compounds **1~3** inhibited the growth of the tested pathogenic bacteria with minimum inhibitory concentrations of 8~32 µg/mL, while compound **3** exhibited antifungal activity against *Magnaporthe oryzae* (IC_50_, 5.21 µg/mL). A comparison of these data with those of the ustilaginoidins provided insights into the structure-bioactivity relationships.

## Introduction

Ustilaginoidins were bis-naphtho-γ-pyrones of 9,9′-linkage produced by the pathogen *Villosiclava virens* (anamorph: *Ustilaginoidea virens*)^1–5^, which was the causal agent of rice false smut that threatened the production of rice worldwide^6,7^. Up to now, 26 ustilaginoidins have been reported from *V*. *virens*, including ustilaginoidins A~J^1–3^, K~P^4^, Q~W^5^, and E_1_^4^, 2,3-dihydroustilaginoidin T^5^, and isochaetochromin B_2_^4,5^. These compounds were considered to be mycotoxins, as they were teratogenic towards mouse embryo limb bud and midbrain cells^8^, and cytotoxic against several cancer cells including KB (epidermoid)^9^, A2780 (ovarian)^4^, HCT116 (colon), NCI-H1650 (lung), BGC823 (gastric), Daoy (medulloblastoma), and HepG2 (liver) cells^5^. The toxicities may attributed to their inhibition of ATP synthesis in mitochondrial^10^. In addition, they showed phytotoxicities on the elongation of radicle or plumule of rice seeds^4,5^, and antibacterial activities^4,11^.

The chirality of ustilaginoidins included not only the axial chirality, which was reported to be a*R*^4,5,12^, but also that of the stereogenic centers in the 2,3-dihydropyran-4-one ring when present, which was, however, unknown with regard to the absolute configurations for most of them. Among the 26 reported ustilaginoidins, 18 of them have one or two 2,3-dihydropyran-4-one moiety (Supplementary Fig. S1), in which the absolute configuration of the chiral center(s) was unknown, except that of ustilaginoidin D^3^. It was worth mentioning that the absolute configuration of ustilaginoidin D was proposed only by comparing the ^1^H, and ^13^C NMR data, melting point, and CD spectra with those of the known analogue, chaetochromin A^3,12^. Theoretically, there could be several solutions to address the absolute configuration problem. One was X-ray crystallographic analysis, which was used to establish the absolute configuration of chaetochromin A^12^, a diastereoisomer of ustilaginoidin D, by analysis of its 5,5′,6′,8,8′-penta-*O*-methyl-6-*O*-*p*-bromobenzoate, however, the difficulties in obtaining suitable crystals in different solvent systems, and insufficient amounts for chemical derivatization followed by crystallization, hindered the use of this method to determine the absolute configuration. Another solution was using the chiroptical spectroscopic method, which has been successfully used in solving the stereochemistry of complex natural products^13^, however, the ECD spectra of chiral biaryl compounds were dominated by its axial chirality, as exemplified by the study of cephalochromin^14^, a diastereoisomer of ustilaginoidin F, thus not suitable to determine the absolute configurations at the stereogenic center(s) of ustilaginoidins. Although Polavarapu, *et al*. succeeded in determining the absolute configuration of cephalochromin by comparing the experimental and theoretical VCD spectra^14^, the vibrational bands used to distinguish the diastereomers were of small magnitudes, and the applicability of this method to the bis-naphtho-γ-pyrones with three or four stereogenic centers (C-2, C-2′, C-3, and/or C-3′) still needed to be verified.

In this study, we addressed the stereochemical problem of ustilaginoidins from a biosynthetic point. Three biosynthetic monomers were obtained from a gene knockout mutant (Δ*UV_2091*), and were evaluated for their biological activities. In addition, the structure-activity relationships were discussed by comparison with those of the dimers (*i*.*e*. ustilaginoidins).

## Results and Discussion

**Figure 1:**
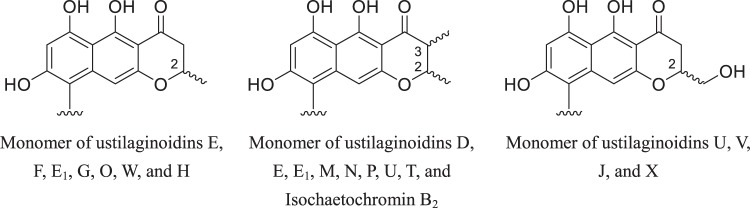
Different substitution patterns of the monomeric building blocks of the ustilaginoidins containing a 2,3-dihydropyran-4-one moiety.

Ustilaginoidins are dimers of naphtho-γ-pyrones, and all have an a*R* configuration for the C-9/9′ axis. For those 18 ustilaginoidins that contain stereogenic center(s) in the 2,3-dihydropyran-4-one ring (Supplementary Fig. S1), only three patterns of substitutions to the ring have been reported so far, that is 2-methyl, 2,3-dimethyl, or 2-hydroxymethyl (Fig. 1). Since the absolute configuration of the axial chirality for these 18 compounds has been determined, the establishment of the absolute configuration of the monomer should allow the construction of the stereochemistry of the whole molecule. In the current study, we succeeded in isolating and identifying the monomers through biosynthetic study.

### *UV_2091* is responsible for the dimerization of naphtho-γ-pyrone monomers

**Figure 2:**
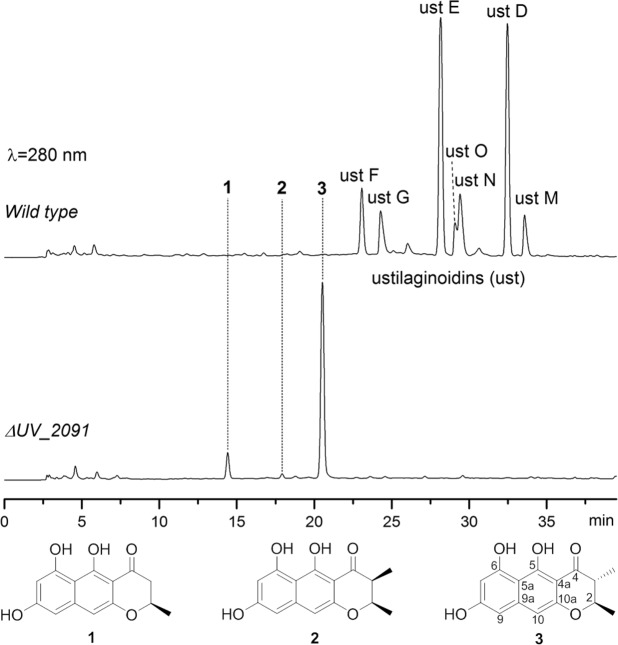
HPLC analysis of the EtOAc extracts obtained from the wild type and the Δ*UV_2091* mutant strain. The peaks observed between 23~34 min in the wild type corresponding to ustilaginoidins F, G, E, O, N, D and M, respectively.

The biosynthesis of chaetochromin A, a diastereoisomer of ustilaginoidin D, has been studied by feeding experiment with isotope-labeled precursors, confirming that the folding pattern of the polyketide chain was the same as that of rubrofusarin^15^. Recently, the whole genome of *V*. *virens* UV8b has been sequenced^16^, and the putative PKS gene cluster for ustilaginoidin biosynthesis was predicted by comparison with that of the aurofusarin (an oxidized product of the dimeric 9-hydroxyrubrofusarin) in *Fusarium graminearum*^17^. In this gene cluster, the genes *UV_2086*, *UV_2087*, *UV_2088* and *UV_2091* were highly homologous to *PKS12*, *FG_02325*, *aurT* and *gip1* in *F*. *graminearum*^17^, respectively. We particularly interested in *UV_2091*, a homology of *gip1*, which was reported to encode a laccase that dimerized two molecules of 9-hydroxyrubrofusarin into the C-7/7′ dimer in *F*. *graminearum*^17^. Thus, we knocked out this gene with the CRISPR-Cas9 system^18^ and obtained the knockout mutant (Δ*UV_2091*), whose colony morphology was obviously different from that of the wild type by having less yellow pigment (Supplementary Fig. S2), hinting the absence or reducing amount of ustilaginoidins. Indeed, HPLC analysis revealed that the ustilaginoidins found in the wild type strain (P1) were absent in the Δ*UV_2091* strain, while three other compounds with more polarity and displaying similar but not ustilaginoidin-type UV absorption (Supplementary Fig. S3) were found in the EtOAc extract of the Δ*UV_2091* strain (Fig. 2). LC-MS analysis disclosed that these three compounds have molecular mass about half of the ustilaginoidins. Scale-up fermentation of the Δ*UV_2091* strain in rice medium, extraction of the resulting fungal mycelia with EtOAc, followed by removal of the solvent under vacuum, a brownish extract was obtained. The EtOAc extract was chromatographed over LH-20 and purified by semi-preparative HPLC, which led to the isolation of three monomers (**1**~**3**) (Fig. 2).

### Structural elucidation

Compound **3** was the major and the most polar metabolite among the isolated monomers. It was isolated as a yellow amorphous powder. In the HRESIMS spectrum, a prominent pseudomolecular peak was observed at *m*/*z* 273.0767 [M-H]^−^, suggesting the molecular formula as C_15_H_14_O_5_. The IR spectrum exhibited absorptions for hydroxyl (3394 cm^−1^), conjugated keto (1631 cm^−1^), and phenyl (1582, 1498 cm^−1^) groups. Its UV spectrum was similar to that of ustilaginoidins, though not identical (Supplementary Fig. S3), suggesting that it contained the same naphtho-γ-pyrone skeleton. The fact that its molecular mass is about half of the ustilaginoidin D and isochaetochromin B_2_ hinted the monomer nature of **3**. This was confirmed by NMR analysis. In the ^1^H NMR spectrum, signals attributable to three aromatic protons including two *meta*-coupled (δ_H_ 6.18, 6.354, each d, *J* = 2.0 Hz) and one singlet (δ_H_ 6.348), two methines (δ_H_ 4.14, 2.66, each dq), and two methyl groups (δ_H_ 1.46, 1.22, each d) were observed (Table 1). The ^13^C NMR spectrum displayed 15 carbon signals that were assignable to one keto group (201.9), ten sp^2^-hybridized carbons, two methines (δ_C_ 79.5, 47.4), and two methyl groups (δ_C_ 20.0, 10.4) (Table 1), by the aid of HSQC spectrum. These data resembled those of ustilaginoidin D^3^, except the presence of one additional aromatic proton (δ_H_ 6.354, d, H-9) that showed *meta-*coupling to H-7 (δ_H_ 6.18, d) in **3**. This allowed us to establish the planar structure of **3** as one half of ustilaginoidin D (Fig. 2), which was verified by HMBC experiment.

**Table 1:** ^1^H and ^13^C NMR data of **1**~**3**.

Position	1 (CD_3_OD)		2 (CD_3_COCD_3_)		3 (CD_3_OD)	
	*δ*_C_, type	*δ*_H_, mult. (*J* in Hz)	*δ*_C_, type	*δ*_H_, mult. (*J* in Hz)	*δ*_C_, type	*δ*_H_, mult. (*J* in Hz)
2	74.7, CH	4.50, ddq (11.0, 3.6, 6.2)	76.4, CH	4.68, qd (6.5, 3.2)	79.5, CH	4.14, dq (10.6, 6.2)
3	44.3, CH_2_	2.78, dd (17.4, 11.0)	45.0, CH	2.82, qd (7.3, 3.2)	47.4, CH	2.66, dq (10.6, 7.0)
		2.70, dd (17.4, 3.6)				
4	199.7, C		203.2, C		201.9, C	
4a	103.2, C		102.27, C		102.5, C	
5	165.5, C		ND^a^		165.2, C	
5a	105.2, C		104.8, C		105.2, C	
6	160.9, C		160.5 C		160.8, C	
7	101.0, CH	6.19, br.s	100.8, CH	6.28, d (2.1)	100.9, CH	6.18, d (2.0)
8	163.4, C		163.2, C		163.2, C	
9	102.5, CH	6.37, d (1.6)	102.33, CH	6.53, d (2.1)	102.3, CH^b^	6.354, d (2.0)
9a	143.9, C		143.6, C		143.8, C	
10	101.9, CH	6.39, s	101.5, CH	6.46, s	101.8, CH^b^	6.348, s
10a	156.7, C		155.8, C		156.4, C	
2-CH_3_	21.2, CH_3_	1.46, d (6.2)	16.7, CH_3_	1.39, d (6.5)	20.0, CH_3_	1.46, d (6.2)
3-CH_3_			9.8, CH_3_	1.21, d (7.3)	10.4, CH_3_	1.22, d (7.0)

^a^ND: not detected.

^b^Assignments within a column may be interchanged.

**Figure 3:**
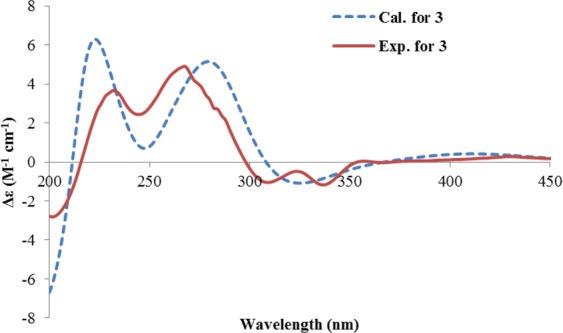
The experimental ECD spectrum of **3**, and the calculated ECD spectrum of (2*R*, *3R*)-**3**.

The large coupling constant (10.6 Hz) between H-2 and H-3 revealed their *trans* relationship. The absolute configuration of **3** was determined by comparing the theoretical and experimental ECD spectra. First, (2 *R*, 3 *R*)-**3** was randomly selected for conformational search using Molecular Merck force field, followed by geometry optimization using the DFT method (B3LYP/6-31 G(d), CPCM = MeOH). Two stable conformers (**3a**, **3b**) were found, with **3a** being the dominating one (91.2%) (Supplementary Fig. S4). Both conformers differed mainly in the γ-pyrone ring, in which 2-CH_3_ and 3-CH_3_ were both equatorial in **3a**, while both axial in **3b**. Both conformers were subjected to TDDFT ECD computations (B3LYP/6-31 + G(d), CPCM = MeOH). Then, the theoretical ECD spectrum of (2*R*, 3*R*)-**3** was generated by averaging that of **3a** and **3b** according to their Boltzmann distributions. The calculated spectrum of (2*R*, 3*R*)-**3** showed negative Cotton effect at 325 nm, and strong positive Cotton effects at 223 and 279 nm, which matched well with the experimental spectrum (Fig. 3). Thus, compound **3** was established as 2*R*, 3*R*, which was consistent with that proposed for ustilaginoidin D^3^. Compound **3** was a new compound, and a trivial name hemiustilaginoidin D was given.

Compound **1** was isolated as the most polar and the second major monomer. Its molecular formula, C_14_H_12_O_5_, as determined by HRESIMS, contained one less CH_2_ unit than that of **3**. Compound **1** has a similar UV, IR, and NMR spectra compared to those of **3**, implying they share a similar skeleton. However, the notable differences of the NMR data (Table 1) were observed at C-3, where the methyl group in **3** was missing, while a methylene group (δ_H_ 2.78, 2.70, each dd; δ_C_ 44.3) in **1** replaced that of the methine in **3**. The HMBC experiment revealed correlations from 2-CH_3_ (δ_H_ 1.46, d) to C-2 (δ_C_ 74.7), and C-3 (δ_C_ 44.3), and from H_2_-3 (δ_H_ 2.78, 2.70) to C-2, C-4 (δ_C_ 199.7), C-4a (δ_C_ 103.2), and 2-CH_3_ (δ_C_ 21.2). Thus, **1** was determined as a 3-demethyl derivative of **3** (Fig. 2).

**Figure 4:**
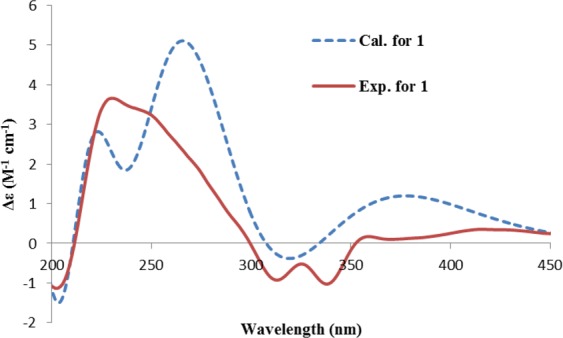
The experimental ECD spectrum of **1**, and the calculated ECD spectrum of (2*R*)-**1**.

The CD spectrum of **1** was similar to that of **3**, however, not like the two positive peaks observed at 232 and 268 nm in **3**, only one peak (230 nm) and a shoulder peak was seen for **1** (Fig. 4). In order to determine the absolute configuration, the ECD spectrum of **1** was calculated. The geometry optimization of the low-energy conformers of (2*R*)-**1**, generated by MMFF conformational search, resulted in only one predominant conformer **1a** (Supplementary Fig. S5). In this stable conformation, the methyl group adopts an equatorial orientation with regard to the dihydropyranone ring. The calculated ECD spectrum of this conformer was comparable to the measured spectrum, thus allowing the assignment of 2*R* configuration of **1**. This structure corresponds to one half of ustilaginoidin F^3^, thus we named it hemiustilaginoidin F.

**Figure 5:**
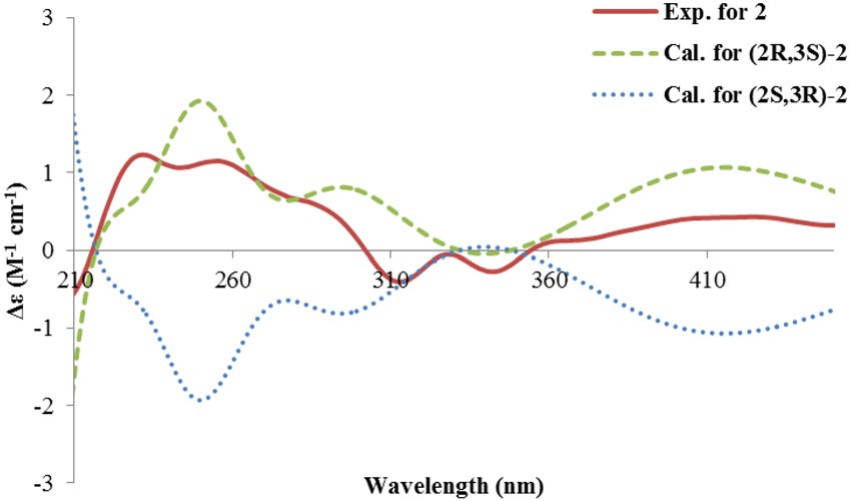
The experimental ECD spectrum of **2**, and the calculated ECD spectra of (2*R*, *3S*)-**2**, and (2*S*, *3R*)-**2**.

Compound **2** was isolated as a minor compound. It had the same molecular formula as that of **3**, indicating that it was an isomer of the latter. A detailed analysis of the NMR data (Table 1) disclosed that they shared a same gross structure. This was confirmed by analysis of the HSQC and HMBC correlations, which also allowed the unambiguous assignment of the ^1^H and ^13^C chemical shifts. The differences between them were attributed to C-2 and C-3, where 2-CH_3_ and 3-CH_3_ were revealed to be *cis*-oriented in **2** from the small coupling constant (3.2 Hz) of ^3^*J*_H-2, H-3_. The CD spectrum of **2** was different from that of **3** mainly in the range of 210~300 nm (Fig. S7). Again, the absolute configuration of **2** was established by comparing the predicted ECD spectrum with the experimental one. (2*R*, 3*S*)**-2** and its enantiomer (2*S*, 3*R*)**-2** were selected for ECD calculations. Two lowest energy conformers were found for (2*R*, 3*S*)**-2**, with the major conformer accounting for 81.5% of the populations, and the other one 18.5%. In the major one, 2-CH_3_ was found to be equatorial, while 3-CH_3_ was axial with regard to the half-chair conformation of the dihydropyranone ring. On the contrary, axial 2-CH_3_ and equatorial 3-CH_3_ were seen in the minor conformer (Supplementary Fig. S6). This was similar for its enantiomer (2*S*, 3*R*)**-2** (Supplementary Fig. S6). The ECD spectrum for each conformer was calculated, then the Boltzmann-averaged ECD spectra for (2*R*, 3*S*)-**2**, and (2*S*, 3*R*)-**2** were generated (Fig. 5). The predicted spectrum for (2*R*, 3*S*)-**2** fitted well with the experimental one, while (2*S*, 3*R*)-**2** did not. Thus, compound **2** has a 2*R*, 3*S* configuration. It is a 3-epimer of **3**, and named epihemiustilaginoidin D.

Comparing the ECD spectra of **1**~**3** revealed similar negative Cotton effects at around 310 and 340 nm, while positive Cotton effects at 210~300 nm though with different peak shape and intensity (Supplementary Fig. S7). It seems that these negative Cotton effects may correspond to the 2*R* configuration for this type of structure.

**Figure 6:**
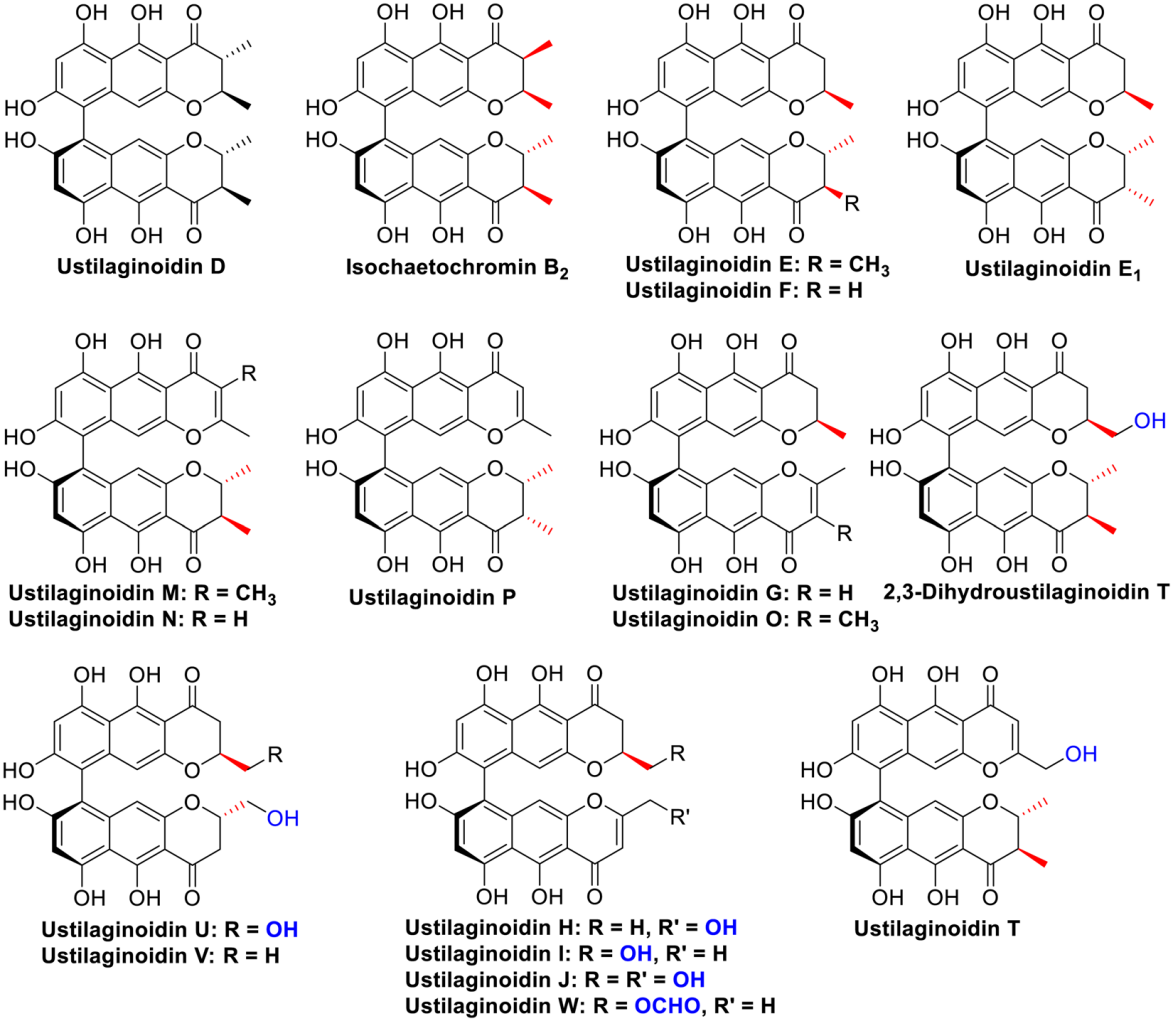
Proposed absolute structures for the ustilaginoidins mentioned in Figure S1.

It was interesting that no 2-hydroxymethylated counterpart of **1** was detected in the EtOAc extract of Δ*UV_2091* by HPLC-DAD-MS analysis. This suggested that 2-methylhydroxylation might happen after dimerization of the monomers to form the 2-hydroxymethyl-containing ustilaginoidins. Nevertheless, the configuration at C-2 should retain for the 2-hydroxymethyl substitution, based on a biogenetic consideration. With this information in hand, the absolute structures of those ustilaginoidins with stereogenic centers at the dihydropyranone ring were constructed for the first time (Fig. 6).

### Phytotoxic, cytotoxic, and antimicrobial activities

In our previous study, ustilaginoidins were found to have phytotoxic, antibacterial, antifungal, and cytotoxic activities^4,5^. In order to compare the bioactivities between the monomer and the dimers, compounds **1**~**3** were evaluated for these activities using the same method.

Compounds **1** and **3** were tested for their phytotoxic activities against the germination of the rice (*Oryza sativa* L.) seeds (Table 2). They dose-dependently inhibited the growth of rice radicles and plumules, with the radicles more susceptible to the tested substances. Compound **1** inhibited rice radicle growth with inhibition ratio in the range of 7.62~53.37% dependent on the tested concentration and rice cultivation varieties (Lijiang or Zhonghua 11), while compound **3** showed inhibition ratio of 21.88~75.24%. The variety Zhonghua 11 was more susceptible to compound **3** than that of Lijiang, whereas no significant difference was found between both varieties with regard to compound **1**. Compound **3** showed stronger inhibition against the plumule elongation for both varieties than that of **1**, and this held true for the inhibitory effects on the radicle growth of the Zhonghua 11 variety, but not for that of the Lijiang variety. In the literature, ustilaginoidins B, E, F, I, O, R and U, and isochaetochromin B_2_ were found to be phytotoxic towards the radicle elongation of rice seeds^4,5^. These ustilaginoidins showed more than 50% inhibition ratio against the growth of radicle for the variety Lijiang at 200 μg/mL^4,5^, while compounds **1** and **3** exhibited inhibition ratio of 52.2% and 49.35%, respectively, at 400 μg/mL, for the same rice variety. Taking account of the monomeric characters of **1** and **3**, they did not showed stronger phytotoxicities than these bioactive dimers (ustilaginoidins). For instance, compound **1** (52.2%, 400 μg/mL) showed weaker inhibition than its homodimer ustilaginoidin F (72.22%, 200 μg/mL)^4^, while compound **3** (49.35%, 400 μg/mL) was weaker than its heterodimer isochaetochromin B_2_ (61.35%, 200 μg/mL)^4^. However, we could not draw a clear structure-activity relationship regarding the monomers and dimers.

**Table 2:** Phytotoxic activities of **1** and **3** on radicle and plumule growth of rice and lettuce seeds.

Compound	Concentration (µg/mL)	Inhibition ratio of radicle growth (%)^a^			Inhibition ratio of plumule growth (%)^a^		
		*O*. *sativa* var. Lijiang	*O*. *sativa* var. Zhonghua 11	*L*. *sativa*	*O*. *sativa* var. Lijiang	*O*. *sativa* var. Zhonghua 11	*L*. *sativa*
**1**	50	22.28 ± 1.03 h	7.62 ± 2.56 h	21.57 ± 2.12 h	9.28 ± 3.37 f	5.45 ± 1.16 e	6.94 ± 2.02 f
	100	30.58 ± 1.80 g	33.89 ± 1.91 g	33.97 ± 2.57 f	10.95 ± 5.73 f	5.45 ± 2.57 e	13.65 ± 0.57 e
	200	35.81 ± 0.66 f	43.81 ± 5.19 f	41.91 ± 3.35 e	14.40 ± 0.85 ef	10.41 ± 1.35 e	19.69 ± 2.01 d
	400	52.20 ± 4.83 d	53.57 ± 1.68 e	100.00 ± 0.00 a	14.43 ± 1.54 ef	11.59 ± 0.71 e	100.00 ± 0.00 a
**3**	50	21.88 ± 0.76 h	51.43 ± 1.35 e	11.47 ± 2.24 j	19.56 ± 5.72 de	21.82 ± 5.14 d	4.88 ± 0.12 f
	100	27.26 ± 2.29 g	55.71 ± 2.02 e	15.82 ± 2.96 i	22.32 ± 0.14 de	34.09 ± 3.21 c	14.44 ± 2.29 e
	200	46.12 ± 1.52 e	62.70 ± 1.12 d	27.26 ± 2.00 g	23.37 ± 6.23 d	35.00 ± 4.50 c	20.53 ± 2.70 d
	400	49.35 ± 1.52 de	75.24 ± 4.04 c	100.00 ± 0.00 a	24.40 ± 2.81 d	35.91 ± 5.79 c	100.00 ± 0.00 a
Glyphosate (positive control)	50	88.47 ± 1.11 c	89.19 ± 0.13 b	75.92 ± 2.80 d	47.15 ± 3.33 c	57.14 ± 3.67 b	18.51 ± 0.77 d
	100	93.45 ± 0.32 b	89.50 ± 0.98 b	77.41 ± 3.59 cd	59.29 ± 0.33 b	59.09 ± 2.57 b	27.63 ± 0.95 c
	200	97.03 ± 3.03 ab	95.43 ± 1.14 a	80.23 ± 1.77 bc	60.73 ± 1.33 a	63.64 ± 1.29 ab	28.80 ± 2.43 c
	400	98.64 ± 0.36 a	98.07 ± 0.17 a	82.97 ± 1.53 b	70.73 ± 1.33 a	68.18 ± 2.57 a	56.81 ± 3.65 b

^a^The values were expressed as means ± SD (n = 3). Different letters indicated significant differences among treatments in each column at *p* ≤ 0.05.

Meanwhile, we also tested the phytotoxic activities of **1** and **3** against the germination of the lettuce (*Lactuca sativa* L. var. *ramose* Hort.) seeds (Table 2). Similarly, the plumule was less susceptible to the tested compounds compared to the radicle. When tested at 50~200 μg/mL, both compounds showed dose-dependent inhibitions against the radicle and plumule elongation (inhibition ratio <50%), with compound **1** showing general stronger effect than that of **3**. However, not like the monocotyledonous plant rice, the growth of lettuce (a dicotyledonous plant) was completely inhibited by compounds **1** and **3** at 400 μg/mL, which was better than the positive control glyphosate. Thus, it was tempting to speculate that ustilaginoidins were phytotoxic against lettuce or the other dicotyledonous plants, though we have not tested yet.

The cytotoxicities of **1** and **3** were evaluated against human carcinoma cells (HCT-116, NCI-H1650, BGC-823, Daoy, and HepG2). Compound **1** moderately inhibited the growth of these cells (IC_50_s 13.2~37.3 μM), while compound **3** was inactive (IC_50_ > 50.0 μM) (Table 3), thus implying the 3-methyl group negatively correlated with the cytotoxicity. In the literature, ustilaginoidins A, D, E and G inhibited the growth of KB cells (IC_50_ 0.42~1.94 μM)^9^, while ustilaginoidins K and L showed inhibition against A2780 cells (IC_50_ 4.18 and 7.26 μM, respectively)^4^. Ustilaginoidins B, C, H, I, J, R, S, V and W were cytotoxic to several cancer cell lines (the same as in current study) with IC_50_ values of 4.06~44.1 μM, but none of them was active against all the tested cells^5^. The structure-activity relationship was elusive regarding the monomers and dimers.

**Table 3:** Cytotoxicities of **1** and **3** (IC_50_, μM).

Compound	HCT116	NCI-H1650	BGC823	Daoy	HepG2
**1**	13.2	25.3	21.4	37.3	13.6
**3**	>50.0	>50.0	>50.0	>50.0	>50.0
Taxol (positive control)	0.0019	1.1	0.000107	0.00504	0.0146

Compounds **1**~**3** were screened for antibacterial activities towards pathogenic bacteria including *Bacillus subtilis*, *Staphylococcus haemolyticus*, *Ralstonia solanacearum*, *Xanthomonas vesicatoria*, *Agrobacterium tumefaciens*, and *Pseudomonas lachrymans* (Table 4). All these compounds were active with MIC values of 8~32 µg/mL, which was comparable to the positive control streptomycin sulfate. Among them, compound **1** showed the strongest inhibition with IC_50_ values of 4.75~10.61 µg/mL. Among the tested bacteria, *R*. *solanacearum* was most susceptible to the tested compounds, while *S*. *haemolyticus* was least susceptible. In the literature, ustilaginoidins D, E, G, and N displayed antibacterial activity with IC_50_ values of 2.29~10.64 µg/mL^4^. It seemed that the monomeric compound showed better inhibition against the tested bacteria than that of the dimers (ustilaginoidins). For examples, compound **1** showed inhibitory activity with MIC values ≤ 32 µg/mL, while its homodimer ustilaginoidin F did not exhibit any effect at 128 μg/mL^4^. Though ustilaginoidin D, the homodimer of compound **3**, showed inhibition with MIC values of 32 μg/mL against four tested bacteria, it was inactive against *P*. *lachrymans* and *R*. *solanacearum* (MIC > 64 μg/mL)^4^, while compound **3** displayed inhibition against all six tested bacteria (MIC 16~32 μg/mL). It was interesting that the heterodimer of **2** and **3**, i.e., isochaetochromin B_2_, was inactive at 128 μg/mL^4^, while **2** and **3** were both active with MIC values ≤ 32 µg/mL.

**Table 4:** Antibacterial activities of **1**~**3**.

Bacterium	MIC/IC_50_ (µg/mL)	Compound			
		1	2	3	Streptomycin sulfate^a^
*A*. *tumefaciens*	MIC	16	16	32	32
	IC_50_	7.44	8.85	12.70	9.70
*B*. *subtilis*	MIC	16	32	32	32
	IC_50_	7.66	10.25	8.66	3.48
*P*. *lachrymans*	MIC	8	16	16	8
	IC_50_	5.75	8.72	7.44	5.59
*R*. *solanacearum*	MIC	8	8	16	8
	IC_50_	4.75	4.21	6.26	2.30
*S*. *haemolyticus*	MIC	32	32	32	32
	IC_50_	10.61	20.99	10.69	6.56
*X*. *vesicatoria*	MIC	16	32	16	16
	IC_50_	8.13	15.37	6.86	6.16

^a^Positive control.

In addition, compounds **1** and **3** were evaluated for inhibitory effects towards the rice blast pathogen *Magnaporthe oryzae*. Compound **3** showed strong inhibition against the spores germination with IC_50_ value of 5.21 µg/mL, which was comparable to the positive control carbendazim (IC_50_, 6.86 µg/mL), while compound **1** was moderately active (IC_50_, 58.54 µg/mL). In literature, the antifungal activities of ustilaginoidins have not been reported. Three related bis-naphtho-γ-pyrones with an a*S* axial chirality, cephalochromin, isoustilaginoidin A and dihydroisoustilaginoidin A, were found to be inactive against the tested fungi^19^.

## Conclusion

The absolute structures of ustilaginoidins were important for studying their structure-activity/toxicity relationship, detoxification, chemical synthesis, biosynthesis, pathogenesis and so on. In the current study, the absolute structures of the ustilaginoidins, whose absolute configurations were unknown regarding the stereogenic centers, were constructed for the first time by analysis of the biosynthetic monomers obtained from a gene knockout mutant of *V*. *virens* (Δ*UV_2091*). These monomers were elucidated as hemiustilaginoidin F (**1**), epihemiustilaginoidin D (**2**), and hemiustilaginoidin D (**3**) by spectroscopic analysis, and their absolute configurations were established by TDDFT ECD computations. The monomeric compounds were evaluated for their phytotoxic, cytotoxic, antibacterial and antifungal effects, and insights were gained for the structure-activity relationship by comparing the bioactivities of the monomeric and dimeric naphtho-γ-pyrones (*viz*. ustilaginoidins). The monomeric compounds seemed to have less phytotoxicity against rice seeds than the dimers, but with better antimicrobial effects against the tested bacteria and fungus. More structures should be evaluated for a better understanding of the relationships. In this study, neither 2-hydroxymethylated monomer nor the related metabolite was detected from the mutant strain (Δ*UV_2091*), thus a further investigation on the biosynthesis of ustilaginoidins was necessary and now under progress.

## Methods

### General experimental procedures

Optical rotations, ultraviolet (UV), and circular dichroism (CD), and infrared (IR) spectra were measured on an automatic polarimeter (Autopol III, Rudolph Research Analytical, Hackettstown, New Jersey), UV/vis spectrophotometer (TU-1810, Beijing Persee General Instrument Co., Ltd., Beijing, China), CD spectrometer (JASCO J-815, JASCO Corp., Tokyo, Japan), and FT-IR spectrometer (Nicolet Nexus 470, Thermo Electron Scientific Instrument Crop., Madison, Wisconsin), respectively. High resolution electrospray ionization mass spectrometry (HRESIMS) spectra were measured on a LC/Q-TOF-MS machine (Agilent Technologies, Santa Clara, CA). ^1^H, ^13^C, and 2D NMR (HSQC, HMBC) spectra were recorded on a Bruker Avance 400 NMR spectrometer (Bruker BioSpin, Zürich, Switzerland). Chemical shifts were expressed in *δ* (ppm) and referenced to tetramethylsilane (the inner standard), while coupling constants in Hertz. HPLC-DAD analysis of the EtOAc extracts was performed on a Shimadzu instrument equipping with a SPD-M20A photodiode array detector (LC-20A, Shimadzu Corp., Tokyo, Japan) using an analytic C_18_ column (250 mm × 4.6 mm i.d., 5 μm; Phenomenex Inc., Torrance, California). The column temperature was set at 30 °C. The mobile phase was composed of methanol (B), and water containing 0.02% TFA (A). A gradient elution program eluting from 60% B to 100% B over 40 min was used, and flow rate was 1.0 mL/min. Semipreparative HPLC separation was done on a Lumtech instrument (Lumiere Tech. Ltd., Beijing, China) equipping with a K-501 pump and a K-2501 UV detector using a Luna-C18 column (250 mm × 10 mm i.d., 5 μm, Phenomenex Inc.), with flow rate of 3 mL/min.

### Strains, plasmids, and culture conditions

The wild strain *V*. *virens* P1 was kindly provided by Prof. Wenxian Sun (College of Plant Protection, China Agricultural University, China). *V*. *virens* strain was cultured on YTD (0.1% yeast extract, 0.1% tryptone and 1% glucose) medium at 28 °C. The vector pCas9-tRp-gRNA was constructed as described^18^. Preparation of protoplast and transformation of *V*. *virens* strains using the PEG-mediated method were performed as described^20^. For transformation selection, G418 (MP Biomedicals, Santa Ana, CA) was added to the medium with the final concentration of 700 μg/ml. The primers used in this study were listed in Table S1 (see Supplementary file).

### Generation of 2091 gene replacement construct and mutants

The gRNA spacer 2091-12 (TGACTGGTCACGCTTCACTT) was designed using the gRNA designer program for the best on-target score^21,22^ and analyzed with the Cas9-off program to predict potential off-targets^23^. The sense and antisense oligonucleotides (Supplementary Table S1) of 2091-12 were synthesized and annealed to generate its gRNA spacer as described^24^. The resulting gRNA spacer was cloned between the two BsmBI sites of pCas9-tRp-gRNA by Golden Gate cloning (New England Biolabs). The 1.17-kb upstream and 1.56-kb downstream flanking sequences of 2091 were amplified with primer pairs of 1 F/2 R and 3 F/4 R, respectively, and fused with the geneticin-resistance (GenR) cassette from pFL2^25^ by double-joint PCR. The resulting PCR products were cloned into the pCas9-tRp-2091-12 vector and then transformed into protoplasts of the wild type strain P1. G418-resistant transformants were screened for deletion of 2091 by PCR with primers 5 F and 6 R, and further verified by PCR with primer pairs 7 F/G855R and 8 R/G856F (Supplementary Fig. S2 and Table S1).

### Fermentation, extraction, and isolation

The mutant strain Δ*UV_2091* was grown on potato dextrose agar (PDA, potato 200 g/L, dextrose 20 g/L, and agar 20 g/L) at 25 °C for 10 days. Then, several agar plugs (5 mm × 5 mm) containing mycelia were transferred to the potato dextrose broth medium (100 ml) that was filled in a Erlenmeyer flask (250 mL). The liquid culture was incubated in a rotatory shaker for 10 days (150 rpm, 28 °C) to produce the inoculum, which was used to inoculate the rice medium (100 g of rice, 110 mL of water, in a 1000 mL flask). The static cultivation was performed on 1.0 kg of rice in total at R.T. in the dark, and last for one month. After harvest, the culture was extracted with EtOAc (5 L × 3) for three times. The EtOAc extract was combined, and the solvent was removed using a rotatory evaporator under reduced pressure to yield a brownish residue (2.5 g).

The EtOAc extract (2.5 g) was subjected to size-exclusion chromatography over Sephadex LH-20 (CH_2_Cl_2_-MeOH, 1:1, v/v) to obtain five fractions (Fr. A~E), among which Fr. D (381 mg) was purified by semi-preparative HPLC eluting with MeOH-H_2_O (0.02% TFA) (70:30, v/v) to yield compound **1** (30 mg, t_R_ 18.0 min), compound **2** (2.3 mg, t_R_ 25.0 min) and compound **3** (200 mg, t_R_ 34.0 min).

Hemiustilaginoidin F (**1**). Yellow amorphous powder; [α]^27^_D_ + 75.6 (*c* 0.125, MeOH); UV(MeOH) λ_max_ (log ε) 235 (4.02), 260 (4.02), 324 (3.68), 338 (3.67), 416 (3.67) nm; ECD (c = 9.65 × 10^−4^ M, MeOH) λ (Δε) 416(1.12), 370 (0.32), 358 (0.54), 338 (−3.26), 326 (−1.66), 312 (−2.92), 230 (11.59), 202 (−3.60) nm; ^1^H NMR (CD_3_OD, 400 MHz), ^13^C NMR (CD_3_OD, 100 MHz) see Table 1; HRESIMS *m*/*z* 259.0611 [M-H]^−^ (calcd for C_14_H_11_O_5_, 259.0612).

Epihemiustilaginoidin D (**2**). Yellow amorphous powder; [α]^27^_D_ + 92.5 (*c* 0.125, MeOH); UV(MeOH) λ_max_ (log ε) 233 (4.03), 266 (4.03), 326 (3.66), 337 (3.65), 423 (3.62) nm; ECD (c = 7.31 × 10^−4^ M, MeOH) λ (Δε) 424 (0.43), 362 (0.12), 342 (−0.27), 328 (−0.05), 312 (−0.40), 256 (1.15), 244 (1.07), 232 (1.23), 206 (−0.68) nm; IR ν_max_ 3389, 2981, 1695, 1637, 1507, 1445, 1385, 1358, 1290, 1153, 1085, 994, 908, 851 cm^−1^; ^1^H NMR (CD_3_COCD_3_, 400 MHz), ^13^C NMR (CD_3_COCD_3_, 100 MHz) see Table 1; HRESIMS *m*/*z* 273.0767 [M-H]^−^ (calcd for C_15_H_13_O_5_, 273.0768).

Hemiustilaginoidin D (**3**). Yellow amorphous powder; [α]^27^_D_ + 78.5 (*c* 0.063, MeOH); UV(MeOH) λ_max_ (log ε) 233 (4.14), 268 (4.15), 324 (3.68), 337 (3.67), 414 (3.65) nm; ECD (c = 1.09 × 10^−3^ M MeOH) λ (Δε) 430 (1.00), 366 (−0.11), 358 (0.14), 336 (−4.25), 324 (−1.75), 308 (−3.77), 268 (17.64), 244 (8.77), 232 (13.23), 202 (−10.15) nm; IR ν_max_ 3394, 2980, 1699, 1631, 1582, 1498, 1430, 1385, 1343, 1132, 1092, 1068, 998, 930, 841, 730 cm^−1^; ^1^H NMR (CD_3_OD, 400 MHz), ^13^C NMR (CD_3_OD, 100 MHz) see Table 1; HRESIMS *m*/*z* 273.0767 [M-H]^−^ (calcd for C_15_H_13_O_5_, 273.0768).

### Computation details

The Molecular Merck force field (MMFF) conformational search and DFT/TDDFT calculations were performed as described previously^26^. ECD spectra of each conformer were plotted by the program SpecDis^27^ using the dipole-length computed rotational strengths with Gauss curves and exponential half-width (σ) of 0.45, 0.4 and 0.3 eV, for **1**~**3**, respectively. The calculated ECD spectra for (2 *R*)-**1**, (2 *R*, 3 *S*)-**2**, (2 *S*, 3 *R*)-**2**, and (2 *R*, 3 *R*)-**3** were generated by summation of the spectra of the lowest energy conformers of each structure using the Boltzmann distributions as weighting factors. The calculated ECD spectra were then compared with the experimental ECD spectra to provide information on the absolute configurations of each structure.

### Phytotoxic assay

The isolated compounds were tested for their phytotoxic activities against lettuce (*Lactuca sativa* L. var. *ramose* Hort.) and rice (*Oryza sativa* L.) as described previously^4^. The experiment was performed in a 24-well plate. Briefly, five 3d-germinated rice seeds or ten 1d-germinated lettuce seeds were sown onto a well, which contained 200 μL of working solution. Compounds **1** and **3** were tested at the concentrations of 50, 100, 200, and 400 μg/mL in distilled water containing 2.5% DMSO, and experiments were done in triplicate. For comparison purpose, *N*-(phosphonomethyl)glycine (glyphosate) was used as the positive control. The seeds were grown in a moist chamber at 25 °C in the dark. After 2~3 days, the length of radicle and plumule of each seed was measured. The inhibition ratio (%) was calculated using following equation: [(*L*s-*L*t)/*L*s] × 100, where *L*s and *L*t was the length of the solvent control and the treated, respectively. Compound **2** was not tested, because of the limited amount.

### Cytotoxic assay

Compounds **1** and **3** were tested for cytotoxicities towards human carcinoma cells, including colon cancer cells (HCT-116), non-small-cell lung carcinoma cells (NCI-H1650), gastric cancer cells (BGC-823), medulloblastoma cell (Daoy), and hepatocellular carcinoma cells (HepG2), using the microculture tetrazolium (MTT) assay as described previously^5^. Taxol was used as the positive control.

### Antibacterial assay

Compounds **1**~**3** were evaluated for antibacterial activities against six human/plant pathogenic bacteria, including *Bacillus subtilis* ATCC 11562, *Staphylococcus haemolyticus* ATCC 29970, *Ralstonia solanacearum* ATCC11696, *Xanthomonas vesicatoria* ATCC 11633, *Agrobacterium tumefaciens* ATCC 11158, and *Pseudomonas lachrymans* ATCC 11921, by the modified broth dilution colorimetric assay^28^. The minimum inhibitory concentration (MIC) and half maximum inhibitory concentration (IC_50_) were determined. Streptomycin sulfate was used as the positive control.

### Antifungal assay

The antifungal activities of compounds **1** and **3** were evaluated by testing against the spore germination of the rice blast pathogen *Magnaporthe oryzae* as described previously^28^.

## Supplementary information

Supplementary information
