# Supplementary material for: Determination of the absolute configurations of the stereogenic centers of ustilaginoidins by studying the biosynthetic monomers from a gene knockout mutant of Villosiclava virens
Source: Sci Rep. 2019 Feb 12;9:1855. doi: 10.1038/s41598-018-37941-5 (PMC6372653; doi:10.1038/s41598-018-37941-5)
Supplement: Supplementary file 1 — Supplementary information [file 41598_2018_37941_MOESM1_ESM.pdf]

Supplementary File

Determination of the absolute configurations of the stereogenic centers of ustilaginoidins by studying the biosynthetic monomers from a gene knockout mutant of *Villosiclava virens*

Daowan Lai[1], Jiajia Meng[1], Dan Xu[1], Xuping Zhang[1] Yafeng Liang[2], Yu Han[2], Cong Jiang[2], Huiquan Liu[2], Chenfang Wang[2], Ligang Zhou[1] & Jin-Rong Xu[2,3]

[1]Department of Plant Pathology, College of Plant Protection, China Agricultural University, Beijing 100193, China. [2]Department of Plant Pathology, College of Plant Protection, Northwest A&F University, Yangling 712100, China. [3]Department of Botany and Plant Pathology, Purdue University, West Lafayette, IN 47907, United States.

Daowan Lai, Jiajia Meng and Dan Xu contributed equally to this work.

Correspondence and requests for materials should be addressed to Ligang Zhou (email: lgzhou@cau.edu.cn) or Jin-Rong Xu (email: jinrong@purdue.edu).

Contents

Figure S1. Structures of the reported ustilaginoidins containing at least one 2,3-dihydropyran-4-one moiety from *Villosiclava virens*. 2
Figure S2. Generation of the Δ*UV_2091* mutant........4
Table S1. Primers used in this study........4
Figure S3. Typical UV spectra of the ustilaginoidins (left) and the monomers (right). 5
Computation Data for 1~3........5
Figure S4. The stable conformers of (2*R*, 3*R*)-**3** with populations greater than 1% calculated from their relative free energies (ΔG). 5
Figure S5. The stable conformers of (2*R*)-**1** with populations greater than 1% calculated from their relative free energies (ΔG). 6
Figure S6. The stable conformers of (2*R*, 3*S*)-**2** and (2*S*, 3*R*)-**2** with populations greater than 1% calculated from their relative free energies (ΔG). 7
Figure S7. ECD spectra of **1-3**. 7
(1D, 2D-) NMR, HRESIMS, CD, and IR Data of 1~3........8
Figure S8. ^{1}H NMR spectrum of **1** (CD_3OD, 400MHz)........8
Figure S9. ^{13}C NMR spectrum of **1** (CD_3OD, 100MHz)........8
Figure S10. HMBC spectrum of **1** (CD_3OD). 9
Figure S11. HRESIMS spectrum of **1**. 9
Figure S12. CD spectrum of **1**........10
Figure S13. ^{1}H NMR spectrum of **2** (CD_3COCD_3, 400MHz). 10
Figure S14. ^{13}C NMR spectrum of **2** (CD_3COCD_3, 100MHz). 11
Figure S15. HMBC spectrum of **2** (CD_3COCD_3)........11
Figure S16. HRESIMS spectrum of **2**. 12
Figure S17. CD spectrum of **2**........12
Figure S18. IR spectrum of **2**. 13
Figure S19. ^{1}H NMR spectrum of **3** (CD_3OD, 400MHz)........13
Figure S20. ^{13}C NMR spectrum of **3** (CD_3OD, 100MHz)........14
Figure S21. HMBC spectrum of **3** (CD_3OD). 14
Figure S22. HRESIMS spectrum of **3**. 15
Figure S23. CD spectrum of **3**........15
Figure S24. IR spectrum of **3**. 16

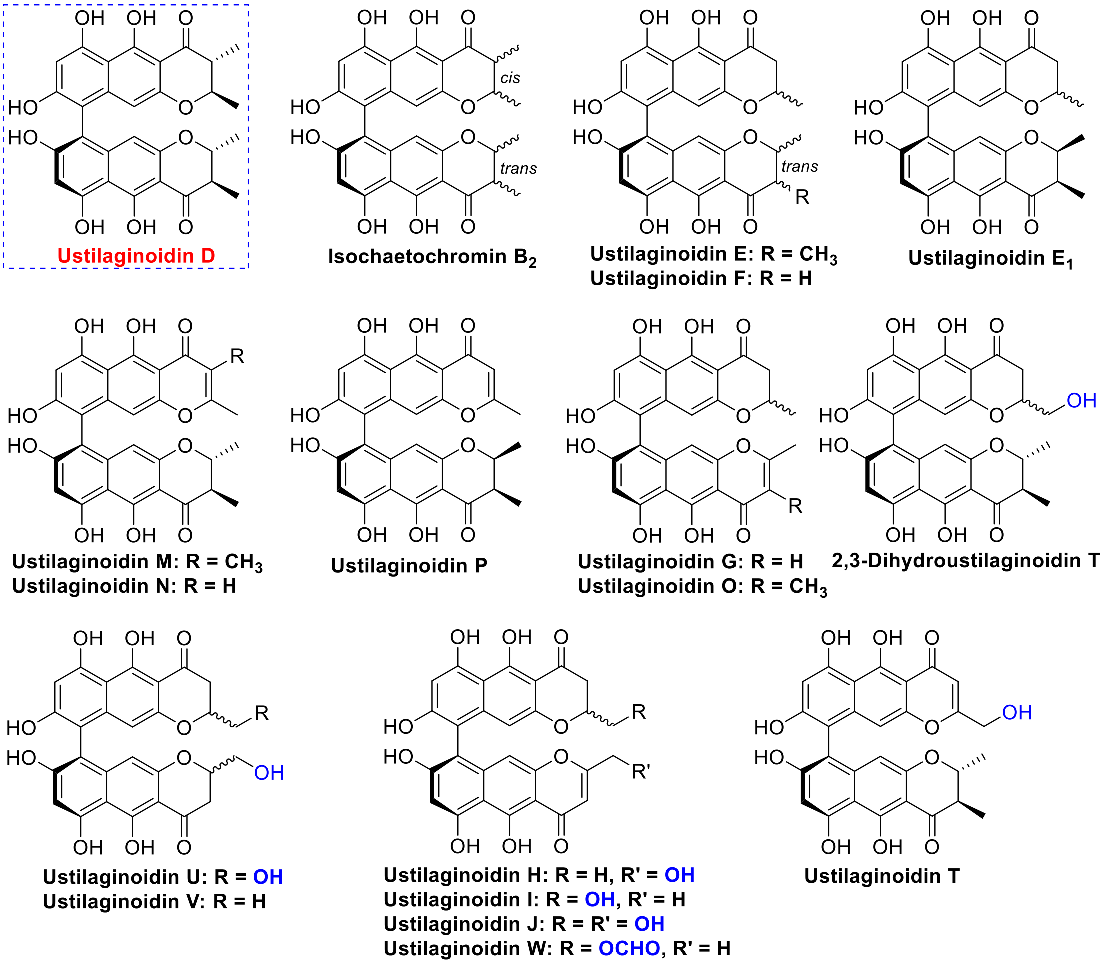

Figure S1. Structures of the reported ustilaginoidins containing at least one 2,3-dihydropyran-4-one moiety from *Villosiclava virens*.

A

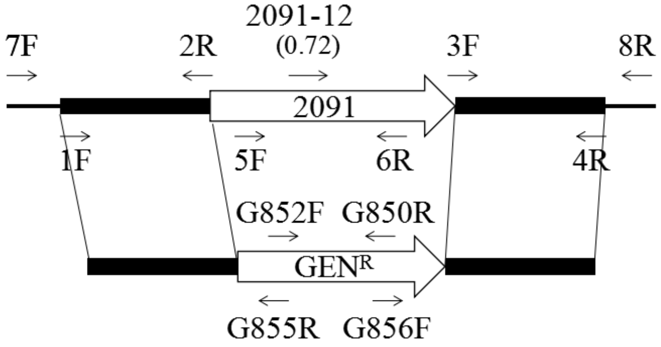

B

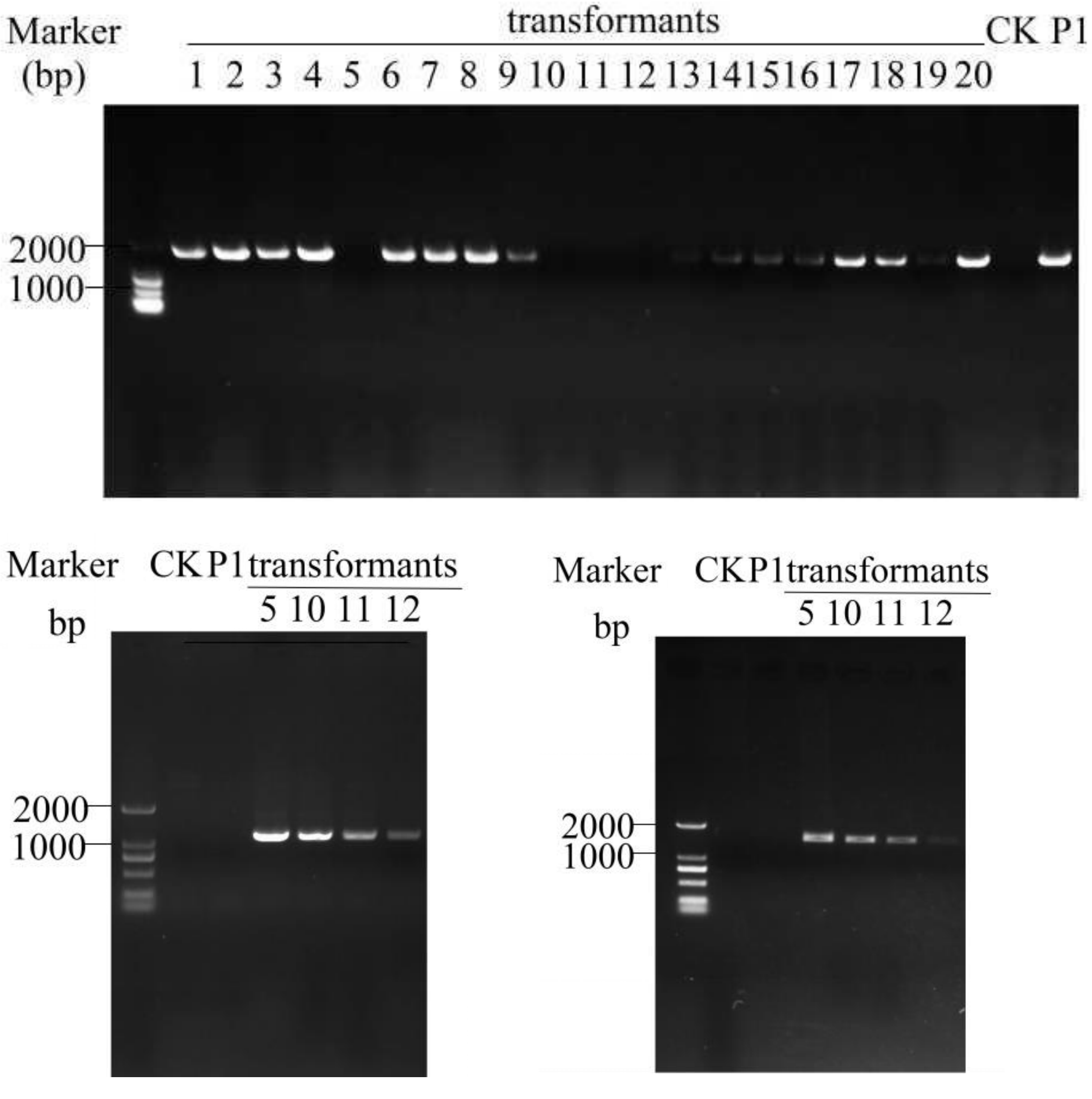

Left Right

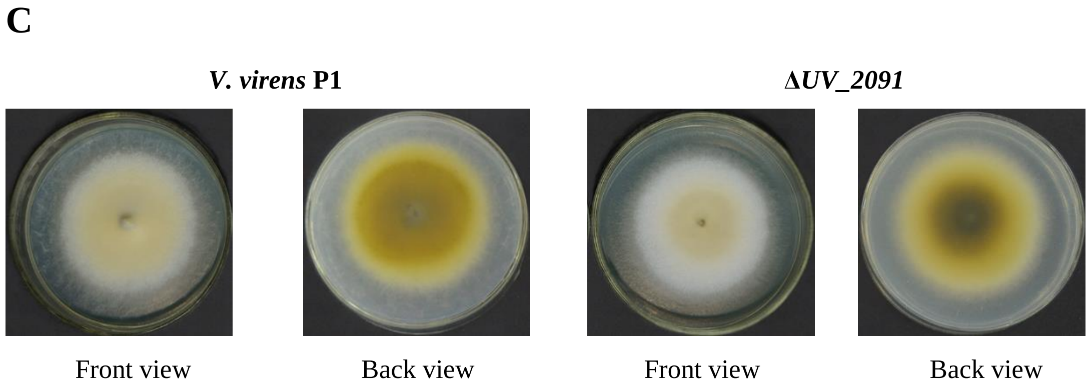

Figure S2. Generation of the Δ*UV_2091* mutant.
(**A**) The 2091 gene and gRNA spacers. The position and direction of gRNA spacers and primers used to generate and screen 2091 deletion mutants are marked with arrows. The on-target scores of spacers 2091-12 is labeled in the bracket. (**B**) PCR assays to verify gene replacement events in 20 transformants. The 1926-bp 2091 fragment was amplified in the wild-type (P1) and 16 transformants with primer 5F/6R. Then 1171-bp upstream (left) and 1568-bp downstream (right) products were amplified with primer pairs 7F/G855R and 8R/G856F in transformants 5, 10, 11, and 12, respectively. Marker: 2 kb DNA ladder marker. H_2O was used as the negative control (CK). (**C**) Colony morphology of the wild type (*V. virens* P1), and Δ*UV_2091* mutant strain grown on PSA plates (28 d).

Table S1. Primers used in this study

2091-12F	acctTGACTGGTCACGCTTCACTT
2091-12R	aaacAAGTGAAGCGTGACCAGTCA
1F	GCTCAGGCACGAGATAAT
2R	CAGATACGGCAGAGAAATCGCAACCTCGAGGCAAGGACAATAGGA
3F	GTTTAGATTCCAAGTGTCTACTGCTGGCGGGCGGCGAGAGAGTAATGT
4R	GCGAAGGAAGGAGAAGGCAC
5F	GCTTCAGATCACGGTCCACA
6R	GAAGTGGCAGTGGAAAAGCC
7F	TACAACCGCCTCTTCCTAC
8R	GGCTTGTAGATTGCGTTCC

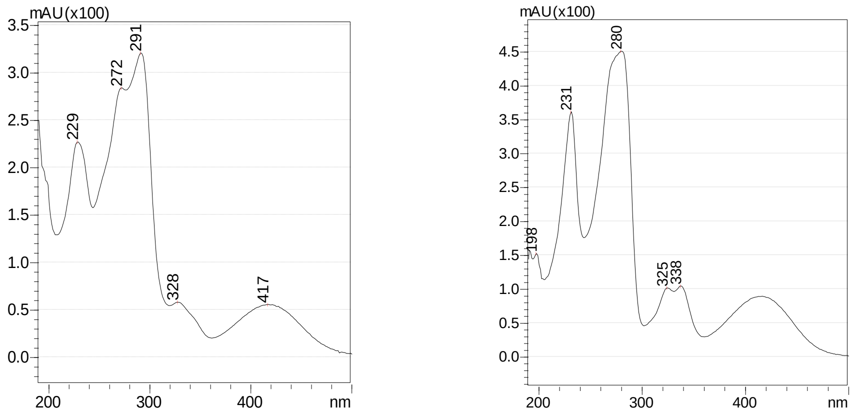

Figure S3. Typical UV spectra of the ustilaginoidins (left) and the monomers (right).

Computation Data for 1~3

3a **3b**

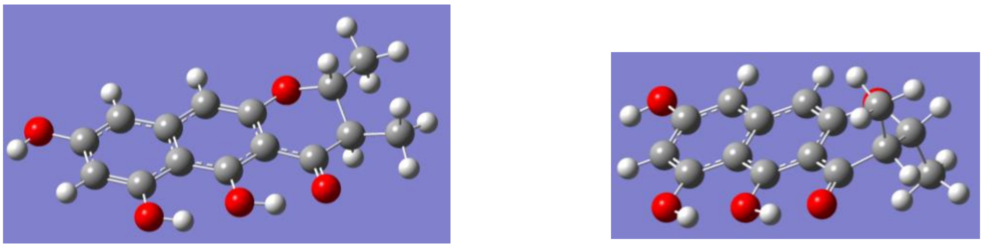

ΔG=0.00 kcal/mol, 91.2% ΔG=1.29 kcal/mol, 8.8%

Figure S4. The stable conformers of (2*R*, 3*R*)-**3** with populations greater than 1% calculated from their relative free energies (ΔG).

1a

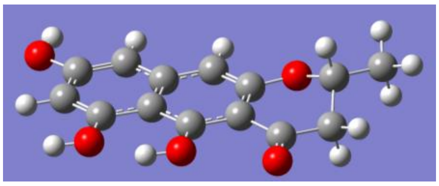

ΔG=0.00 kcal/mol, >99.9%

Figure S5. The stable conformers of (2*R*)-**1** with populations greater than 1% calculated from their relative free energies (ΔG).

(2*R*, 3*S*)-**2**:

(2*R*, 3*S*)-**2a**

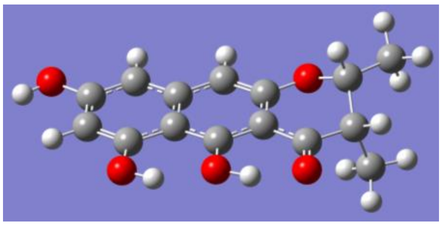

ΔG=0.00 kcal/mol, 81.5%

(2*R*, 3*S*)-**2b**

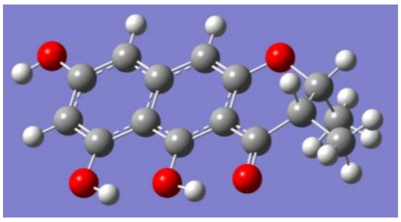

ΔG=0.91 kcal/mol, 18.5%

(2*S*, 3*R*)-**2**:

(2*S*, 3*R*)-**2a**

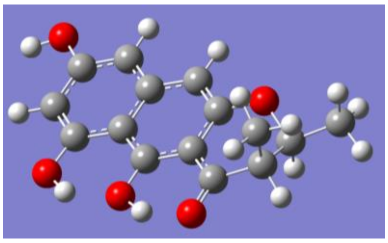

ΔG=0.00 kcal/mol, 81.5%

(2*S*, 3*R*)-**2b**

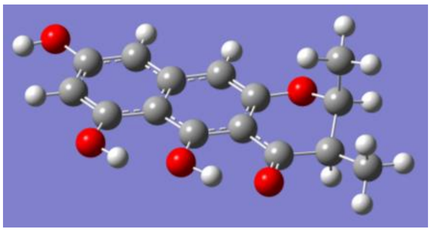

ΔG=0.91 kcal/mol, 18.5%

Figure S6. The stable conformers of (2*R*, 3*S*)-**2** and (2*S*, 3*R*)-**2** with populations greater than 1% calculated from their relative free energies (ΔG).

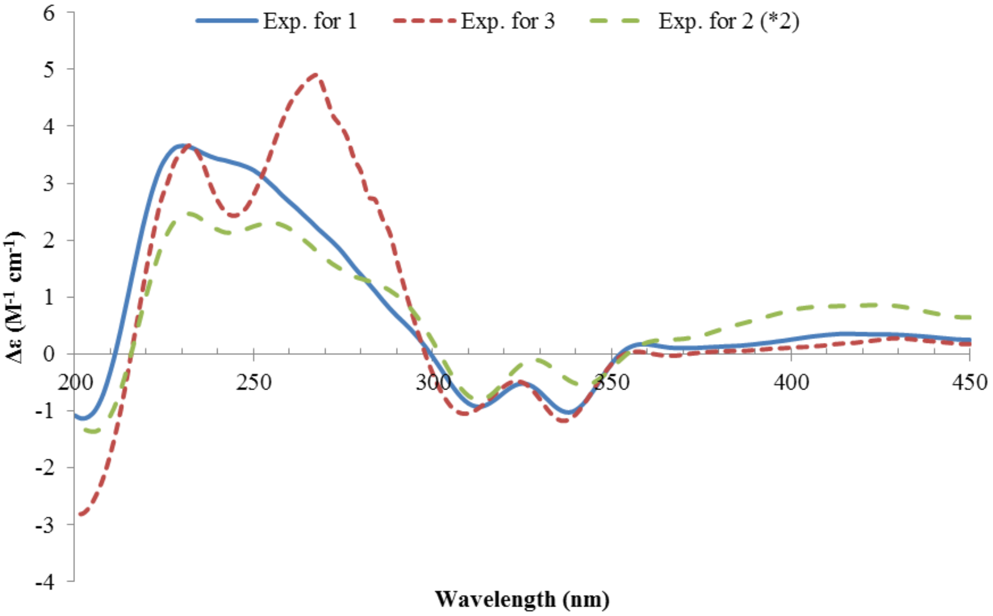

Figure S7. ECD spectra of **1-3**.

(1D, 2D-) NMR, HRESIMS, CD, and IR Data of 1~3

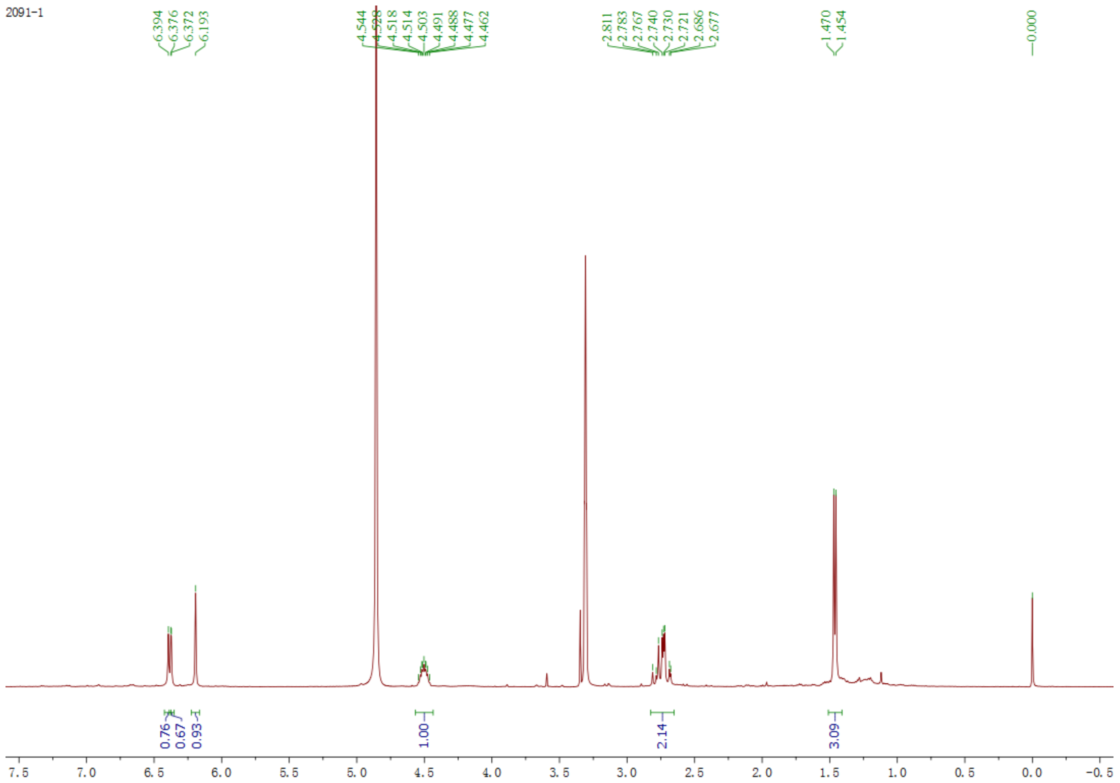

Figure S8. ^{1}H NMR spectrum of **1** (CD_3OD, 400MHz).

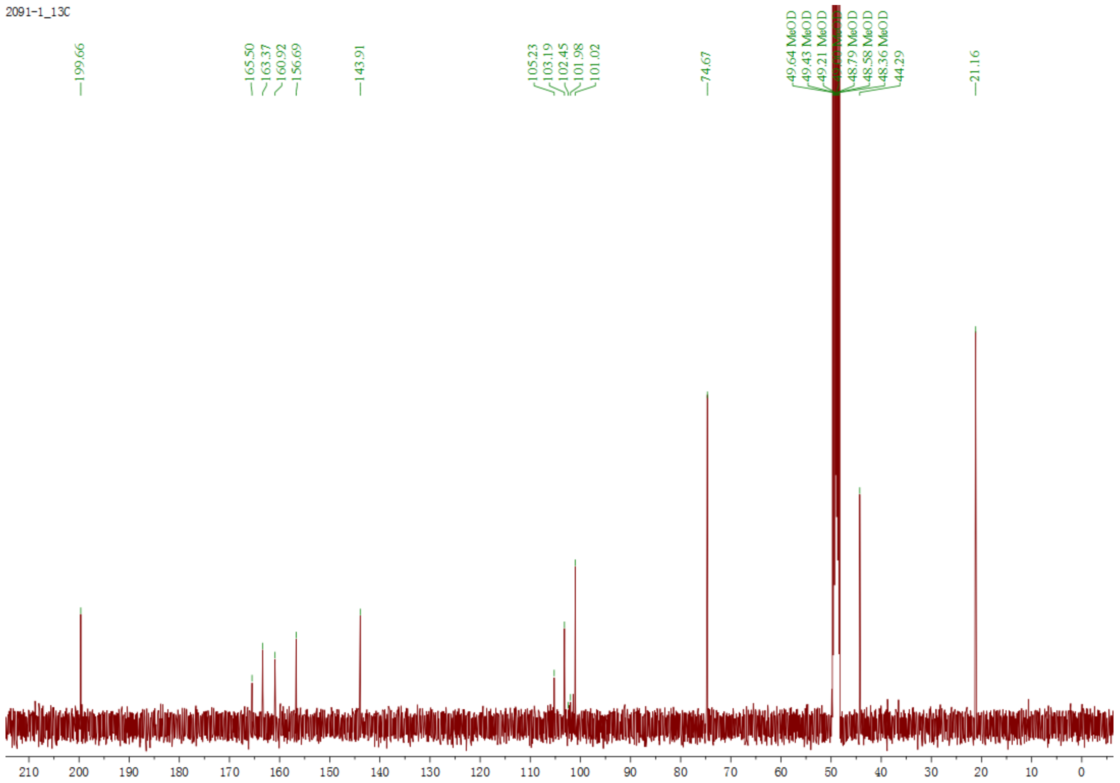

Figure S9. ^{13}C NMR spectrum of **1** (CD_3OD, 100MHz).

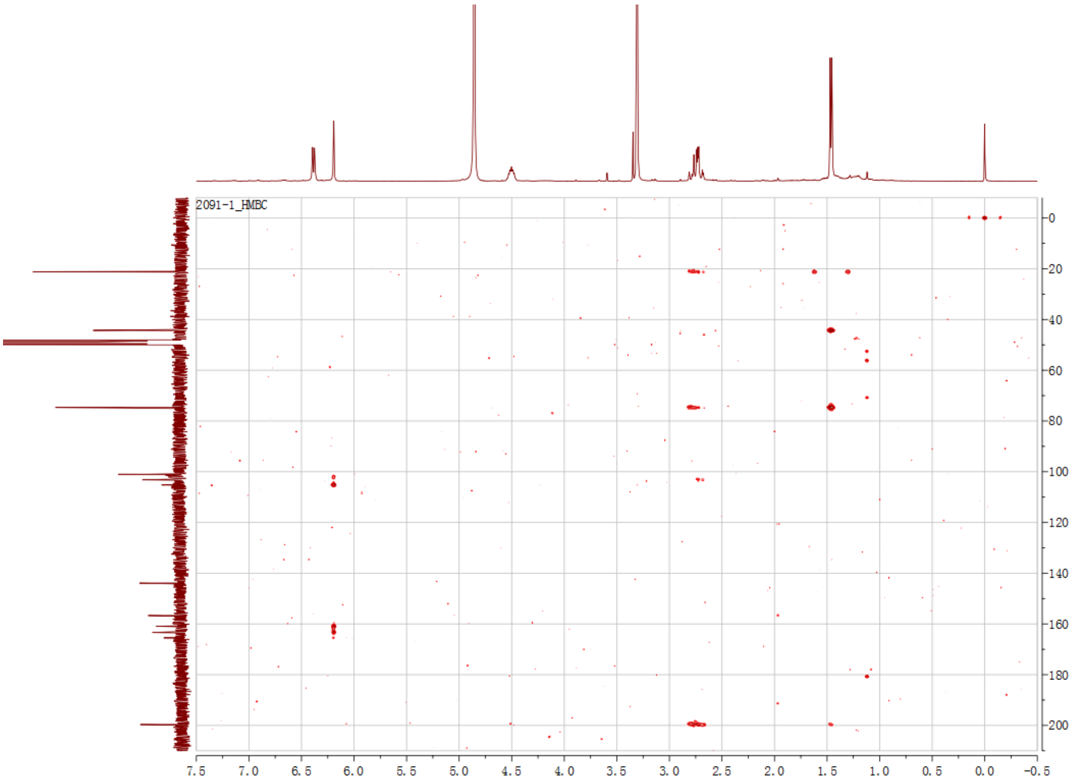

Figure S10. HMBC spectrum of **1** (CD_3OD).

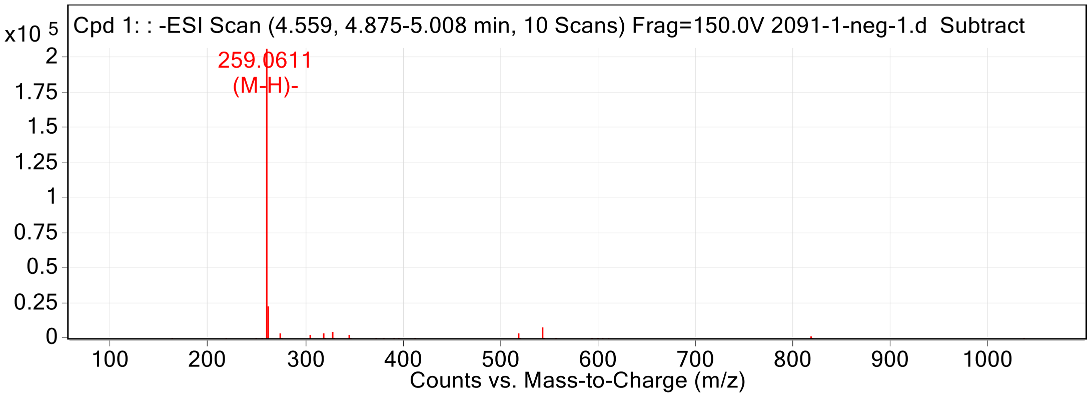

m/z	*Calc m/z*	Diff(ppm)	z	Abund	Formula	Ion
259.0611	259.0612	-0.28	-1	206604.5	$C_{14}H_{11}O_5$	$(M-H)^-$

Figure S11. HRESIMS spectrum of **1**.

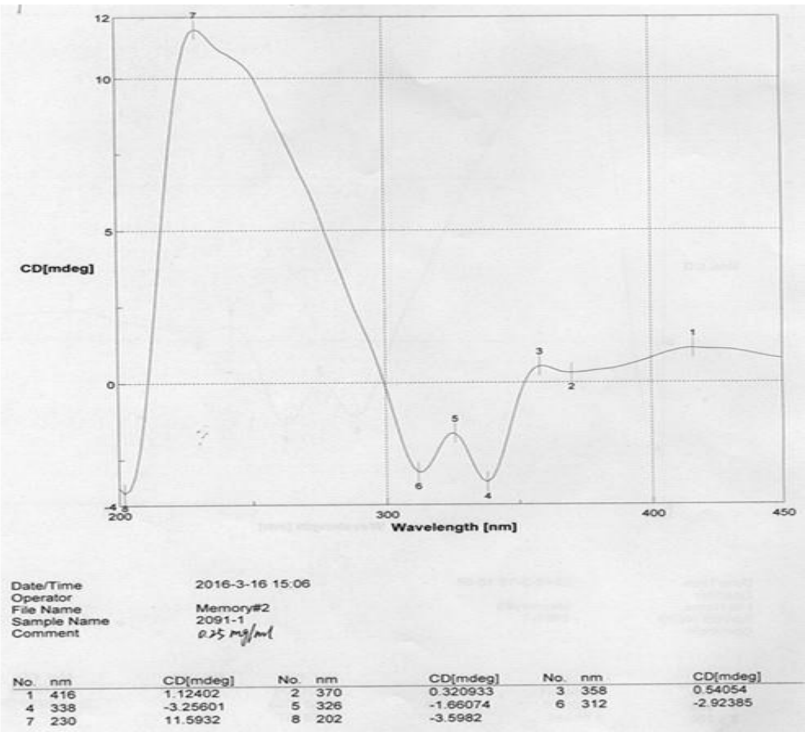

Figure S12. CD spectrum of **1**.

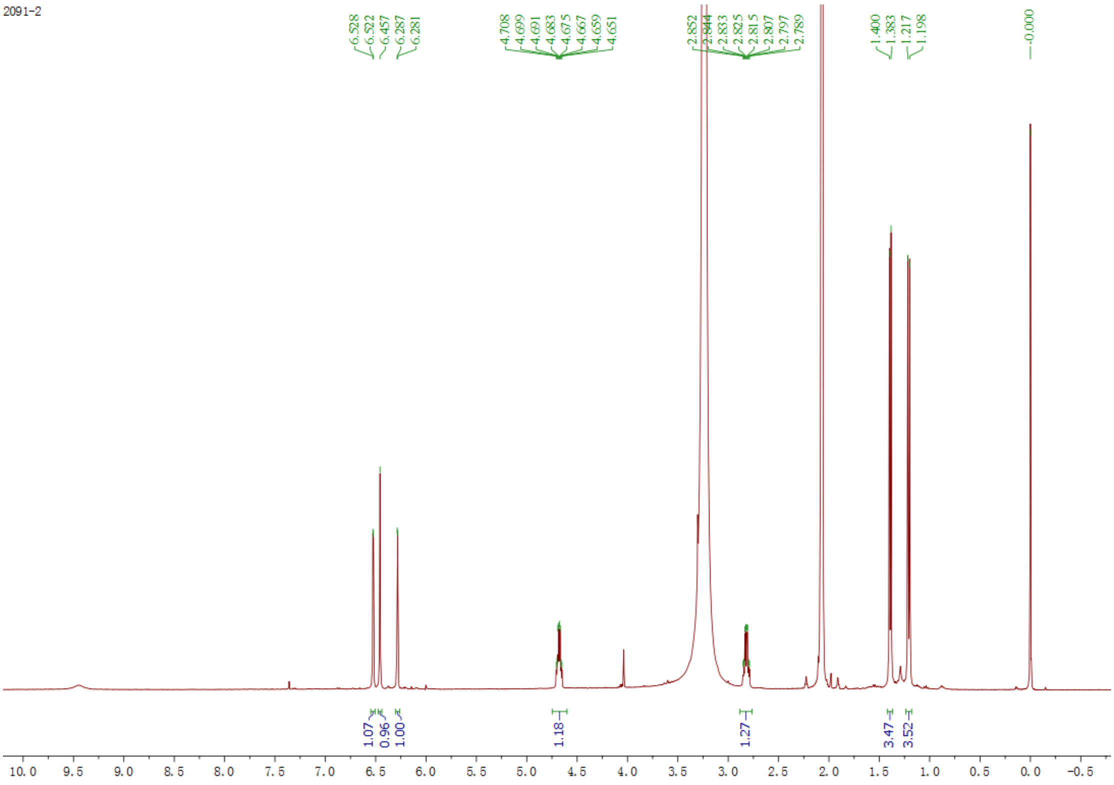

Figure S13. ^{1}H NMR spectrum of **2** (CD_3COCD_3, 400MHz).

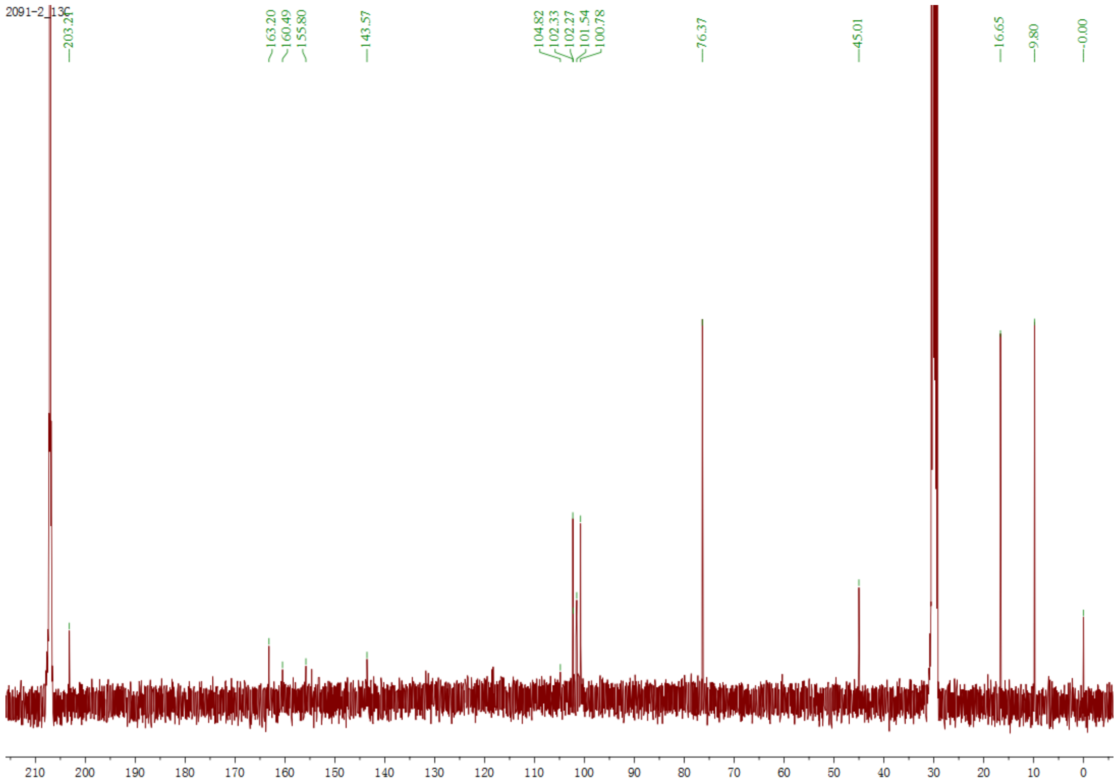

Figure S14. ^{13}C NMR spectrum of **2** (CD_3COCD_3, 100MHz).

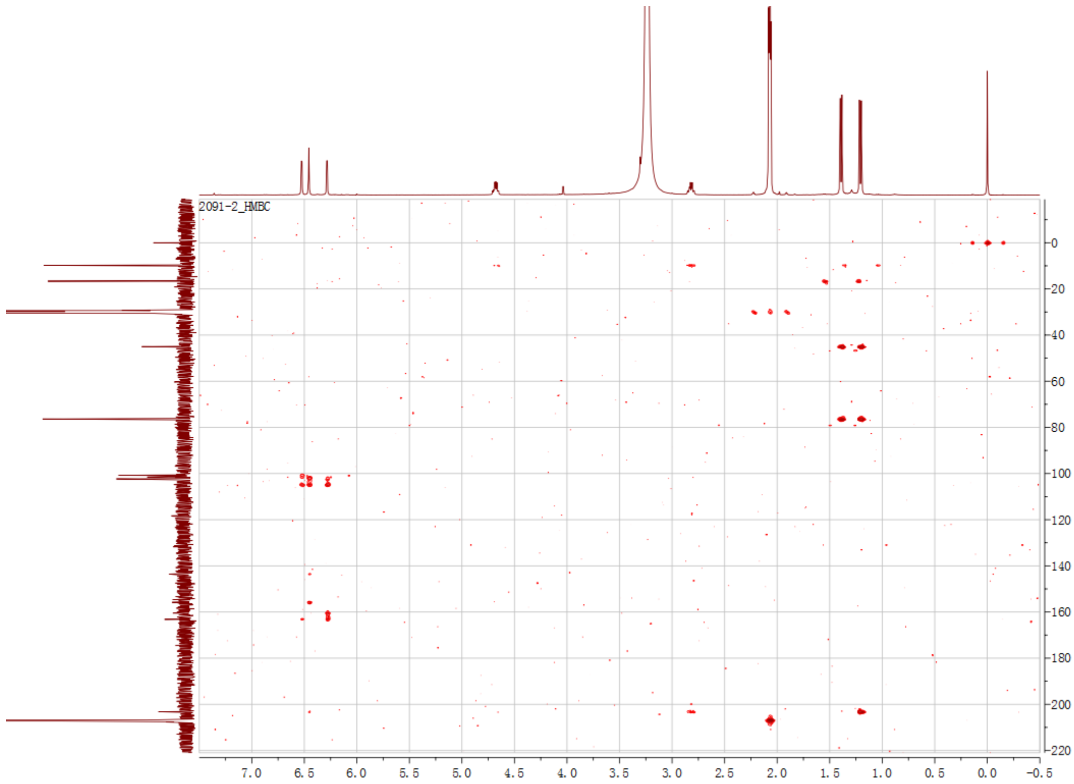

Figure S15. HMBC spectrum of **2** (CD_3COCD_3).

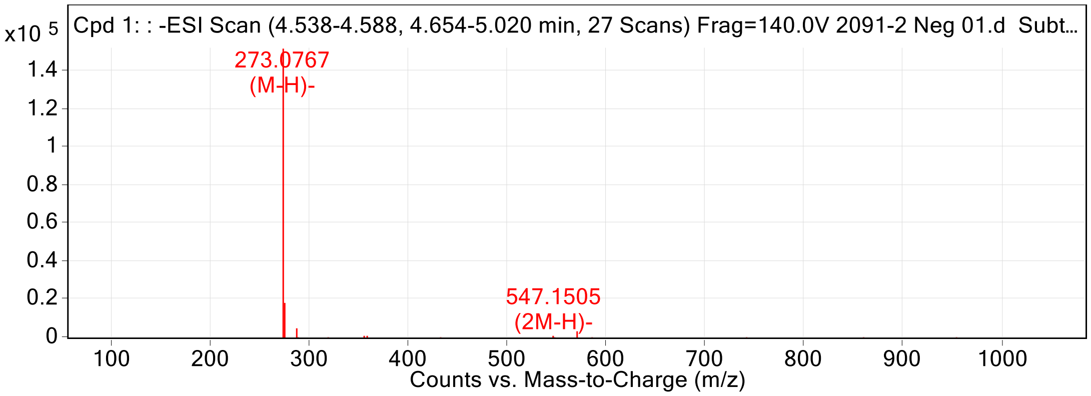

m/z	*Calc m/z*	Diff(ppm)	z	Abund	Formula	Ion
273.0767	273.0768	-0.5	-1	151807.7	$C_{15}H_{13}O_5$	$(M\text{-}H)^-$

Figure S16. HRESIMS spectrum of **2**.

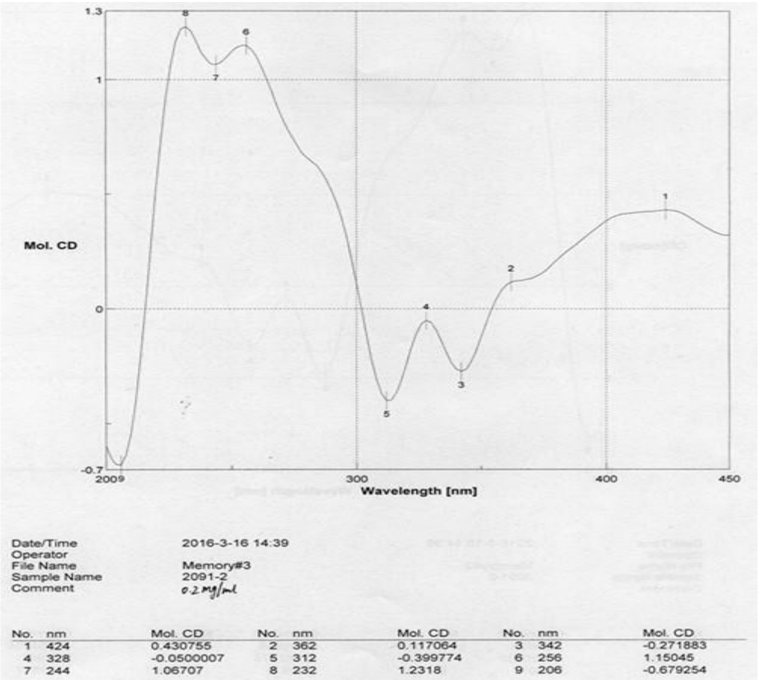

Figure S17. CD spectrum of **2**.

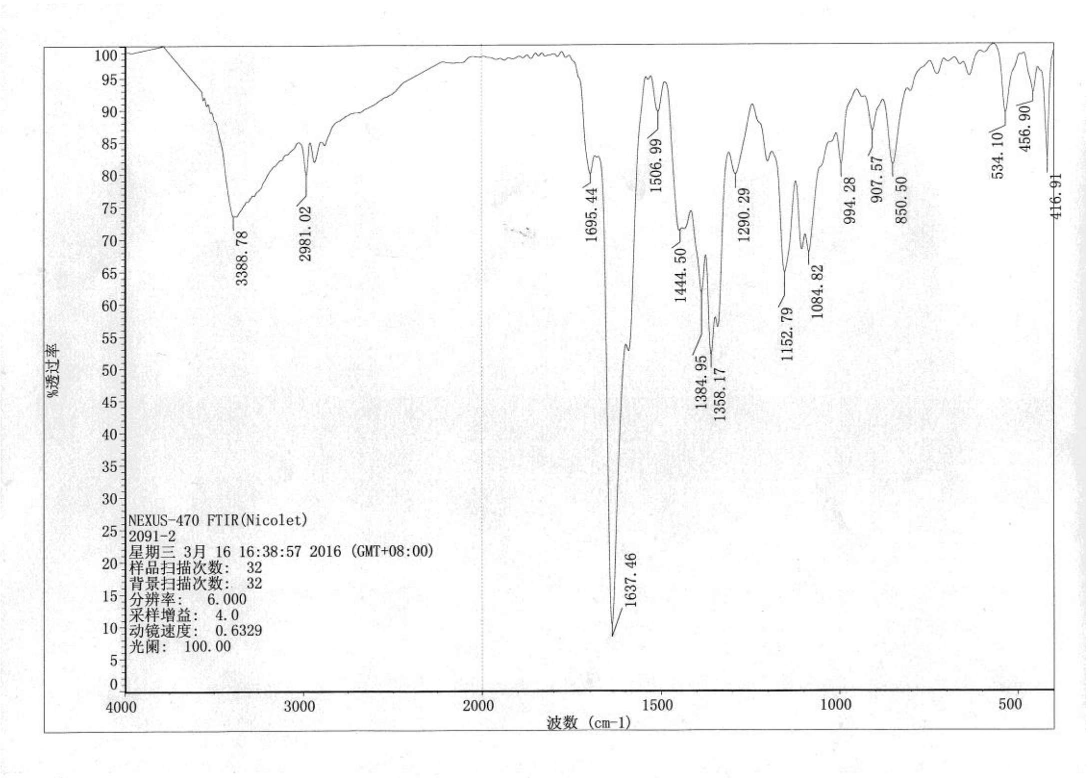

Figure S18. IR spectrum of **2**.

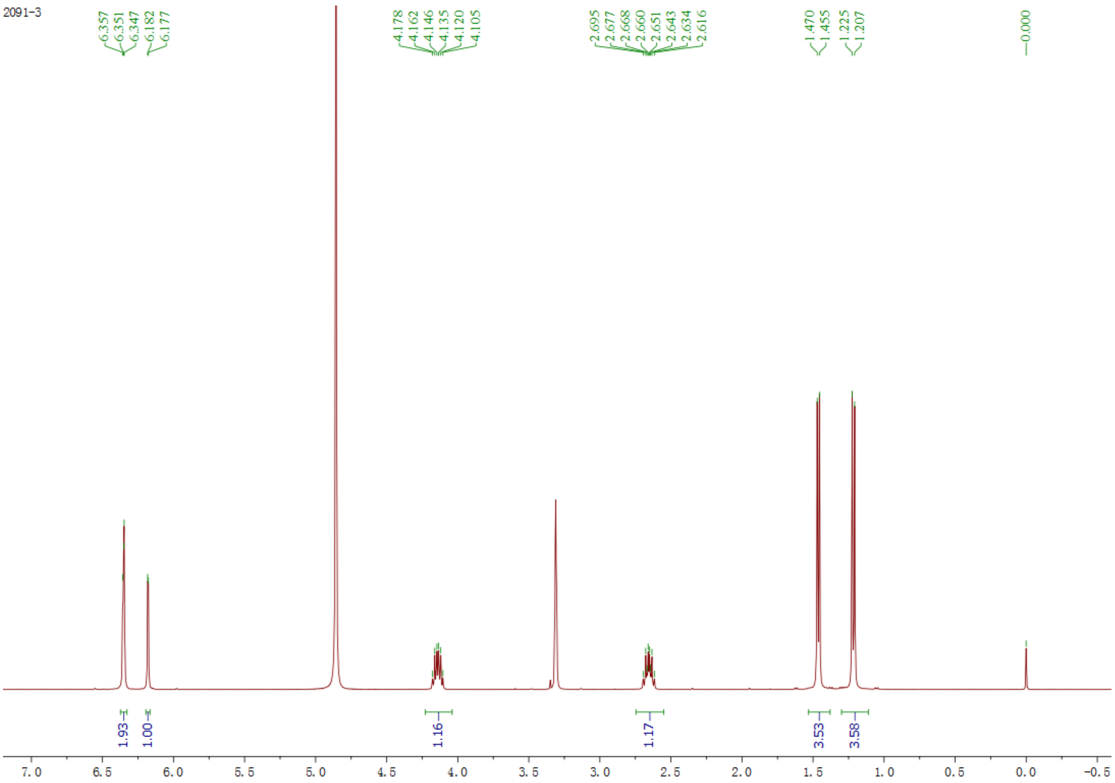

Figure S19. ^{1}H NMR spectrum of **3** (CD_3OD, 400MHz).

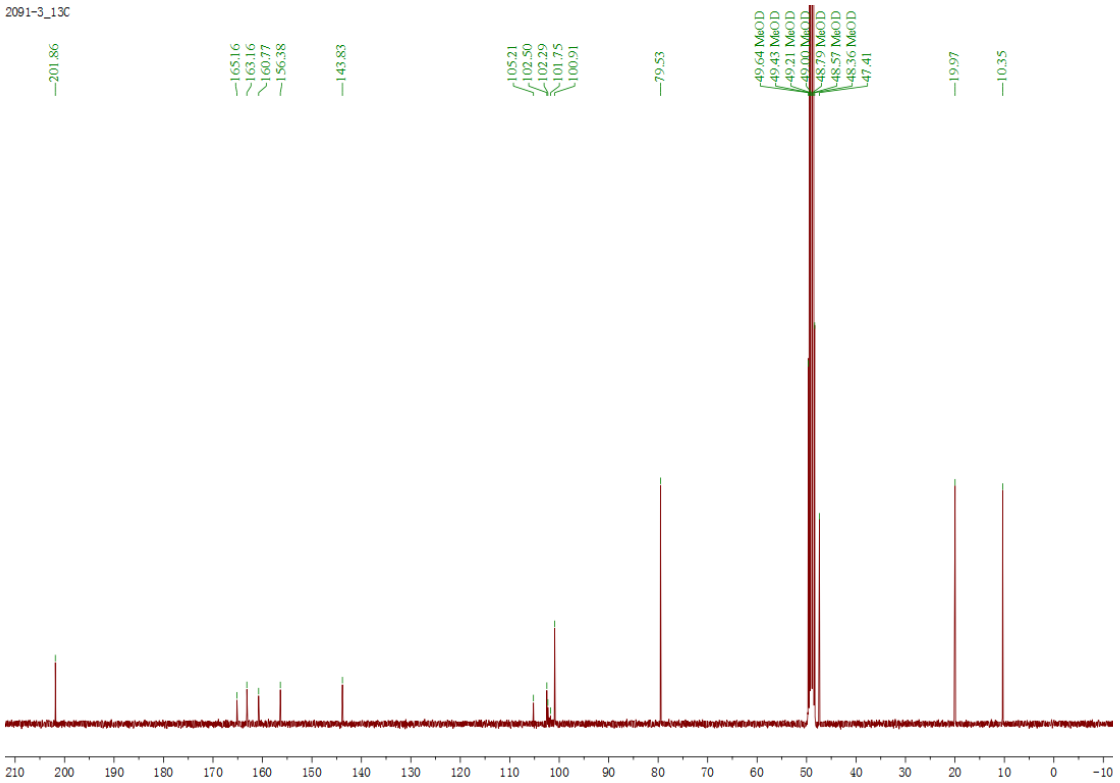

Figure S20. ^{13}C NMR spectrum of **3** (CD_3OD, 100MHz).

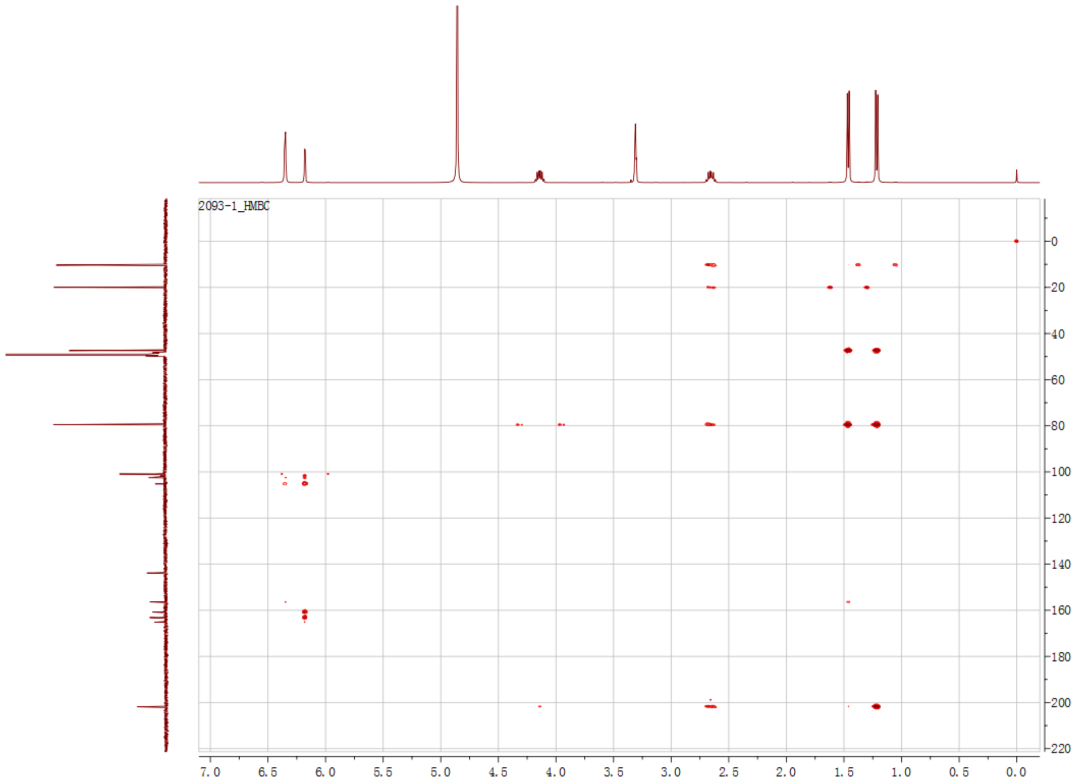

Figure S21. HMBC spectrum of **3** (CD_3OD).

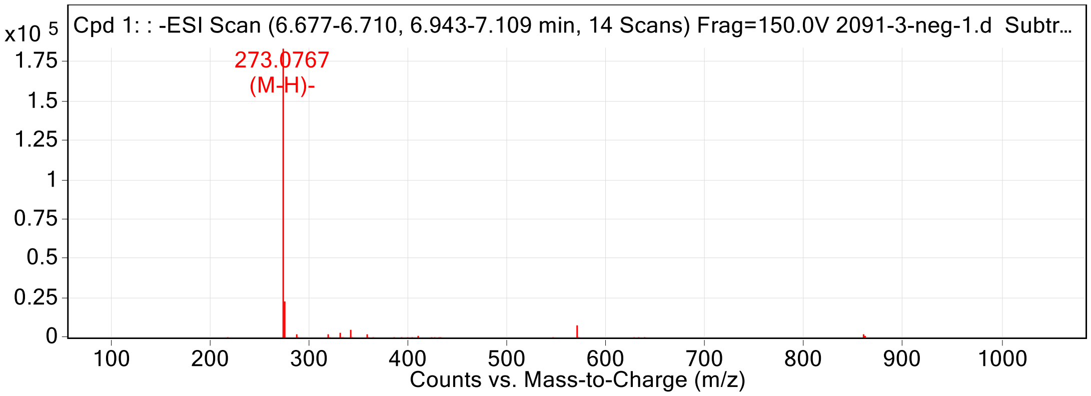

m/z	*Calc m/z*	Diff(ppm)	z	Abund	Formula	Ion
273.0767	273.0768	-0.65	-1	183902.4	$C_{15}H_{13}O_5$	$(M-H)^-$

Figure S22. HRESIMS spectrum of **3**.

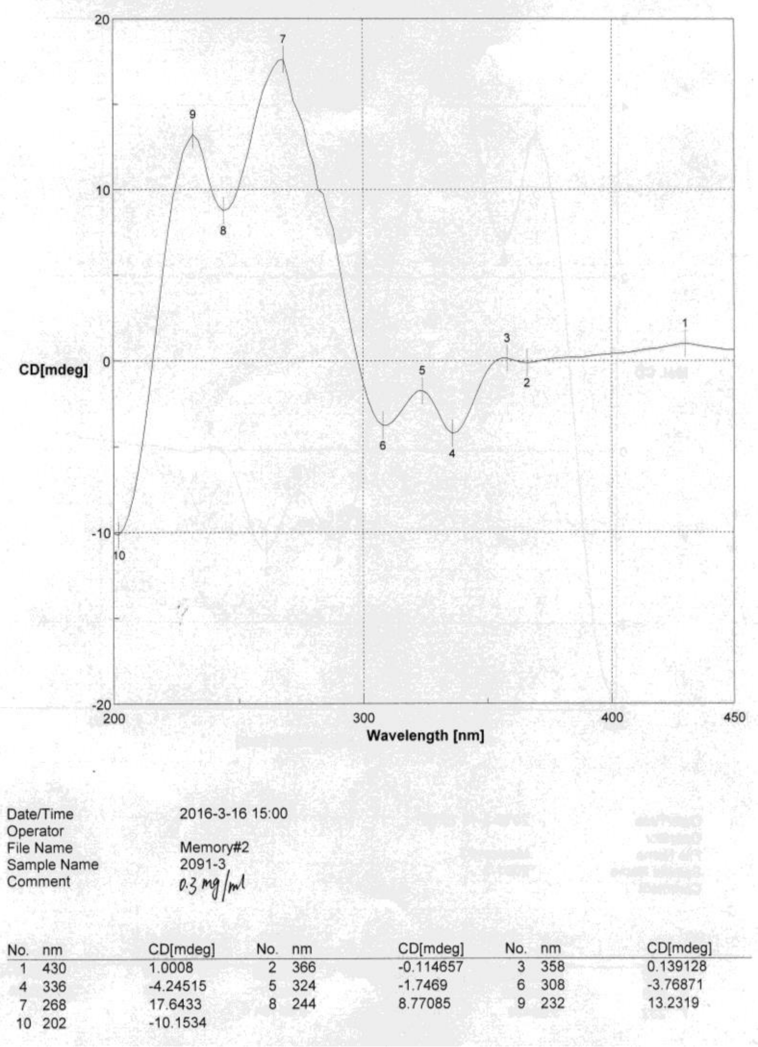

Figure S23. CD spectrum of **3**.

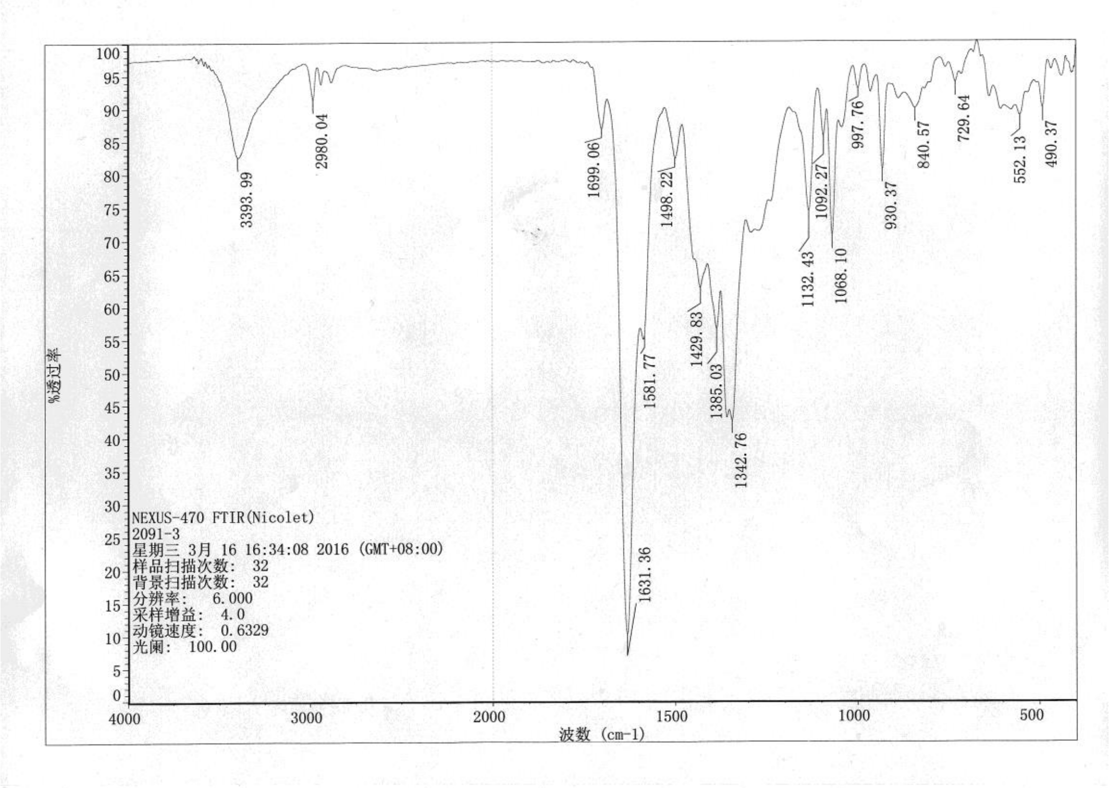

Figure S24. IR spectrum of **3**.
